# The 2019 mathematical oncology roadmap

**DOI:** 10.1088/1478-3975/ab1a09

**Published:** 2019-06-19

**Authors:** Russell C Rockne, Andrea Hawkins-Daarud, Kristin R Swanson, James P Sluka, James A Glazier, Paul Macklin, David A Hormuth, Angela M Jarrett, Ernesto A B F Lima, J Tinsley Oden, George Biros, Thomas E Yankeelov, Kit Curtius, Ibrahim Al Bakir, Dominik Wodarz, Natalia Komarova, Luis Aparicio, Mykola Bordyuh, Raul Rabadan, Stacey D Finley, Heiko Enderling, Jimmy Caudell, Eduardo G Moros, Alexander R A Anderson, Robert A Gatenby, Artem Kaznatcheev, Peter Jeavons, Nikhil Krishnan, Julia Pelesko, Raoul R Wadhwa, Nara Yoon, Daniel Nichol, Andriy Marusyk, Michael Hinczewski, Jacob G Scott

**Affiliations:** 1 Department of Computational and Quantitative Medicine, Division of Mathematical Oncology, City of Hope National Medical Center, Duarte, CA 91010, United States of America; 2 Precision Neurotherapeutics Innovation Program, Mayo Clinic, Phoenix, AZ 85054, United States of America; 3 School of Mathematical and Statistical Sciences, Arizona State University, Tempe, AZ 85281, United States of America; 4 Intelligent Systems Engineering, Indiana University, Bloomington, IN 47408, United States of America; 5 Biocomplexity Institute, Indiana University, Bloomington, IN 47408, United States of America; 6 Oden Institute for Computational Engineering and Sciences, The University of Texas at Austin, Austin, TX 78712, United States of America; 7 Centre for Tumour Biology, Barts Cancer Institute, Queen Mary University of London, London EC1M 6BQ, United Kingdom; 8 Inflammatory Bowel Disease Unit, St. Mark’s Hospital, London HA1 3UJ United Kingdom; 9 Department of Ecology and Evolutionary Biology, University of California, Irvine, CA 92697, United States of America; 10 Department of Mathematics, University of California Irvine, Irvine, CA 92697, United States of America; 11 Department of Systems Biology, Columbia University, New York, NY 10032, United States of America; 12 Department of Biomedical Engineering, University of Southern California, Los Angeles, CA 90089, United States of America; 13 Department of Integrated Mathematical Oncology, H. Lee Moffitt Cancer Center and Research Institute, Tampa, FL 33647, United States of America; 14 Department of Radiation Oncology, H. Lee Moffitt Cancer Center and Research Institute, Tampa, FL 33647, United States of America; 15 Department of Cancer Physiology, H. Lee Moffitt Cancer Center and Research Institute, Tampa, FL 33647, United States of America; 16 Center of Excellence for Evolutionary Therapy, Integrated Mathematical Oncology Department, Moffitt Cancer Center, Tampa, FL33612, United States of America; 17 Department of Computer Science, University of Oxford, Oxford OX1 3QD, United Kingdom; 18 Department of Translational Hematology and Oncology Research, Cleveland Clinic, Cleveland, OH 44195, United States of America; 19 School of Medicine, Case Western Reserve University, Cleveland, OH 44106 United States of America; 20 Evolutionary Genomics and Modelling Lab, Centre for Evolution and Cancer, The Institute of Cancer Research, London SM2 5NG, United Kingdom; 21 Department of Physics, Case Western Reserve University, Cleveland, OH 44106, United States of America; 22 Author to whom any correspondence should be addressed.

**Keywords:** mathematical oncology, mathematical modeling, modeling and simulation, cancer, systems biology, computational oncology

## Abstract

Whether the nom de guerre is Mathematical Oncology, Computational or Systems Biology, Theoretical Biology, Evolutionary Oncology, Bioinformatics, or simply Basic Science, there is no denying that mathematics continues to play an increasingly prominent role in cancer research. Mathematical Oncology—defined here simply as the use of mathematics in cancer research—complements and overlaps with a number of other fields that rely on mathematics as a core methodology. As a result, Mathematical Oncology has a broad scope, ranging from theoretical studies to clinical trials designed with mathematical models. This Roadmap differentiates Mathematical Oncology from related fields and demonstrates specific areas of focus within this unique field of research. The dominant theme of this Roadmap is the personalization of medicine through mathematics, modelling, and simulation. This is achieved through the use of patient-specific clinical data to: develop individualized screening strategies to detect cancer earlier; make predictions of response to therapy; design adaptive, patient-specific treatment plans to overcome therapy resistance; and establish domain-specific standards to share model predictions and to make models and simulations reproducible. The cover art for this Roadmap was chosen as an apt metaphor for the beautiful, strange, and evolving relationship between mathematics and cancer.

## Graphical Abstract

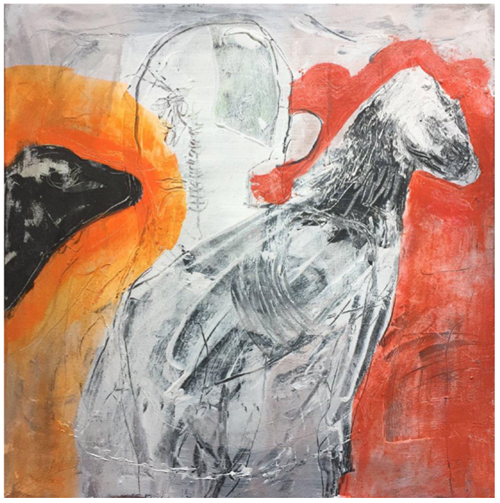

‘Two Beasts’ (2017)

Artist: Ben Day Todd. Acrylic, gouache on canvas ’Two Beasts’ is an exploration of form and colour. By adding and subtracting paint, pushing and pulling colour, new information is found and previously unknown dialogues are recorded.

## 1. Introduction to the 2019 Mathematical Oncology Roadmap

Russell C Rockne^1^

^1^ Department of Computational and Quantitative Medicine, Division of Mathematical Oncology, City of Hope National Medical Center, Duarte, CA 91010, United States of America

Mathematical Oncology—defined here simply as the use of mathematics in cancer research—has gained momentum in recent years with the rapid accumulation of data and applications of mathematical methodologies. The purpose of this 2019 Mathematical Oncology Roadmap is to provide a forward-looking view of the field and to demonstrate specific areas of focus within this unique field of research. The topics presented here are not intended to be exhaustive, but rather to feature emerging, high-impact areas that have the potential to shape the direction of Mathematical Oncology in the next 5–10 years. The selected topics cover both theoretical and practical issues.

The dominant theme of this Roadmap is the personalization of medicine through mathematics, modelling, and simulation. This is achieved primarily through the use of patient-specific clinical data. In this Roadmap, mathematical approaches are used to: make individualized predictions of response to therapy; present data and simulation standards with the goal of creating reproducible models; and improve cancer screening to detect cancer earlier. These approaches are also used to predict and steer cancer evolution to guide the design of adaptive, patient-specific treatment plans that overcome therapy resistance, with the goal of turning incurable cancers into chronic, manageable conditions rather than fatal diseases. Each contribution is summarized here in the order it appears:

### Personalizing medicine by merging mechanistic and machine learning models

The role of Mathematical Oncology in the future of precision or personalized medicine is demonstrated through patient-specific mathematical modelling, analysis of patient-specific clinical data, and patient-specific adaptive therapies. Hawkins-Daarud and Swanson demonstrate these principles by looking towards a future merging of mathematical modelling and machine learning, in which knowledge-based mechanistic modelling is used to guide and inform machine learning when data is sparse. Hawkins-Daarud and Swanson highlight the potential and the challenges of merging these fields of mathematical modelling and machine learning with an application to primary brain cancers and clinical imaging data such as MRI.

### Setting data and model standards

However successful a modelling or simulation method may be, if it cannot be deployed or used by other groups, it is of limited value. For Mathematical Oncology to achieve its highest impact, Sluka *et al* argue that standards are needed for both data and mathematical models, to ensure interoperability, to leverage and build upon prior work, and ultimately to develop useful tools that can be used to study and treat cancer. Of course, the use of standards in science is not new, however, data and model standardization in this domain face unique challenges, particularly with respect to spatial models. Sluka *et al* identify the central challenges and potential advances afforded by the establishment of ‘*FAIR*’ (Findable, Accessible, Interpretable, and Reusable) models in Mathematical Oncology.

### Turning tumour forecasting into a rigorous predictive science

In addition to the challenges of developing and standardizing mathematical models of cancer growth and response to therapy, lies the ‘grand challenge of Mathematical Oncology’: to faithfully reproduce—and predict—the spatiotemporal dynamics of tumour growth. Similar to weather models that predict the path of a hurricane, Hormuth *et al* call for the use of families of models in which the optimal model (or models) is selected with Bayesian methodologies and used to update patient-specific predictions over time. The goal of this approach is to establish a foundation for tumour forecasting as a rigorous predictive science through careful model selection and validation.

### Modelling cancer screening and early detection

Benjamin Franklin famously stated that ‘An ounce of prevention is worth a pound of cure’. Nowhere could this be more true than in cancer; however, nowhere else could this sentiment be more challenging to implement. Many serious issues face the field of early detection of cancer, including the risk of false negatives, false positives, and the possibility of transient early-stage cancers that are successfully defeated by the body’s immune system. However, Curtius and Al Bakir propose that mathematical models of carcinogenesis can be used to evaluate and predict the efficacy of screening strategies using multiscale approaches, with the ultimate goal of producing clinically actionable personalized cancer screening recommendations.

### Analysing cancer dynamics and the evolution of resist ance

As cancer cells grow into a malignant lesion or tumour, the cells evolve and accumulate mutations in their DNA. The analysis of evolutionary dynamics using mathematical models is a rich field that has many applications to cancer. Wodarz *et al* identify the spatial structure of the tumour cell population as a critical challenge in modelling tumour evolution. In particular, they suggest that novel computational methodologies are required to simulate and predict tumour evolution at realistically large population sizes with realistically small rates of mutation. Here, Wodarz *et al* use mathematical modelling to predict the evolution of resistant cells within the evolving cancer as a whole.

### Applying a single-cell view to cancer heterogeneity and evolution

In contrast to the view taken by Wodarz *et al*, Aparicio *et al* consider tumour evolution at single-cell resolution. Using single-cell genome sequencing data, Aparicio *et al* present mathematical and computational methods to analyse single-cell data from a topological perspective. Low-dimensional projections, or visualisations, that are used to study high-dimensional single-cell sequencing data may give a misleading representation of the relationships between individual cells. Aparicio *et al* use machine learning and algebraic topology to construct simplified skeleton graphs as approximations for the geometry of high-dimensional data. These sophisticated methodologies enable the examination of the heterogeneity of individual cells in a continuum of states, from normal/healthy to cancerous. The mathematics of topological data analysis combined with single-cell sequencing technologies provide a powerful tool to study fundamental aspects of cancer biology at an unprecedented resolution.

### Accurately representing metabolism in cancer progression

Altered metabolism and metabolic reprogramming are hallmarks of cancer and are associated with cancer progression and therapeutic resistance. Due to the many interconnected metabolites, enzymes, regulatory mechanisms, and pathways, systems biology approaches have been used to study cell metabolism. Often, mathematical representations of cell metabolism use a constraint-based formalism that does not explicitly account for spatial-temporal variations. Finley proposes a multiscale approach to modelling kinetics and time-varying heterogeneities that may arise in aberrant cell metabolism in cancer due to environmental fluctuations. She also proposes the use of patient-specific data and open source computational platforms that support data and model standards, with the ultimate goal of using these models to generate novel drug combinations and treatment strategies.

### Modelling and predicting patient-specific responses to radiation therapy

Long before the rise of immunotherapy, the three pillars of cancer treatment were surgery, chemotherapy, and radiation therapy. Radiation remains a definitive and curative treatment for many cancers and is highly personalized, with radiation fields and doses sculpted to an individual patient’s anatomy and cancer. However, Enderling *et al* show that radiation therapy outcomes may be predicted and improved using simple mathematical models that account for the growth rate of the cancer and introduce the ‘proliferation-saturation index (PSI)’. The authors discuss challenges for the clinical adoption of this mathematically-defined, patient-specific, predictive response index, and consider the road ahead, which includes prospective randomized clinical trials.

### Pioneering evolutionary therapy

In contrast to the optimization of radiation therapy, Anderson and Gatenby propose an entirely new pillar of cancer treatment: evolutionary therapy. In this paradigm, treatment schedule and dose are mathematically designed to reduce the possibility of treatment resistance. Instead of using the maximum tolerated dose, evolutionary therapy aims to give the minimum effective dose through repeated treatment cycles to maintain tumour control over extended periods of time. Early results from an evolutionary therapy clinical trial in prostate cancer, designed by the authors with in silico ‘phase i’ trials, suggest that the length of treatment cycles is highly patient-specific and may be predicted with mathematical modelling.

### Exploring fitness landscapes and evolutionary game theory

The principles of evolutionary therapy and therapeutic resistance can be modelled mathematically using evolutionary game theory (EGT), in which evolution is determined by selection or optimisation of ‘fitness’. A fitness landscape is a conceptual and mathematical abstraction that enables predictions and interpretations of the temporal process of evolution. However, significant practical and theoretical challenges prevent the measurement or inference of the exact geometry of the fitness landscape. Kaznatcheev *et al* propose that we reconsider the very concept of abstraction itself in order to better understand and use the EGT framework to guide evolutionary therapy, using algorithmic computer science as a practical example.

In contrast to the theoretical considerations of Kaznatcheev *et al*, Krishnan *et al* demonstrate a practical method to experimentally estimate the parameters of an EGT model, with the goal of designing combination therapies that not only avoid therapeutic resistance but are even able to steer cancer evolution on a patient-specific basis. The authors hypothesize—and demonstrate—how EGT-driven therapies can be practically implemented in the clinic to overcome therapeutic resistance in cancer treatment.

### Summary

In summary, this 2019 Mathematical Oncology Roadmap identifies three critical milestones along the path to mathematically designed cancer treatment: (1) obtaining accurate, rigorous, and reproducible predictions of the spatial-temporal progression of cancer; (2) avoiding and mitigating therapeutic resistance; and (3) merging mechanistic knowledge-based mathematical models with machine learning. Surprisingly, despite the emergence of the era of ‘big data’, we are learning that we still lack the right kind of data. Big data in cancer is often taken from a single point in time and space, from only one biological scale, or without an appropriate micro-environmental context. The road ahead includes continued development of knowledge-based mathematical models and methods to bridge big data to the ideal of personalized, predictive, adaptive therapy.

As we look towards the next 5–10 years in Mathematical Oncology, we note that government agencies such as the federal drug administration (FDA) in the United States have begun to officially recognize modelling and simulation as forms of valid scientific evidence in the review and approval process. From our perspective, with the support and adoption of government regulatory agencies that recognize these methodologies, tumour forecasting, patient-specific adaptive therapies with the use of in silico treatment scenarios, virtual clinical trials, and mathematical modelling and simulation have the potential to accelerate our scientific progress in cancer research, and have the potential to transform the way we detect and treat cancer in the clinic.

## 2. The future of personalization in mathematical oncology: a mathematical merger of mechanistic and machine learning models

Andrea Hawkins-Daarud^1^ and Kristin R Swanson^1,2^

^1^ Mayo Clinic, Phoenix, Arizona

^2^ Arizona State University, Tempe, Arizona

### Status

Cancer patient care is intrinsically multidisciplinary. Tumor board is the clinical environment used to bring together those different disciplines to make treatment decisions, but, unfortunately, it is an agonizing environment of doubt—Is the tumor progressing? Is the optimal treatment A or B? All it takes is a brief experience in a tumor board to feel the injustice of a cancer diagnosis and the frustration of not knowing the truly optimal treatment. Every patient is unique and every patient will respond differently to the same treatment protocol. Thus, the central tumor board challenge is—how do we best integrate the unwieldy multitude of dispersed data (imaging, tissue, blood, molecular) to generate optimal clinical decisions for each patient? The current strategy for grappling with this complexity is to average over cohorts of seemingly similar patients with similar diagnoses to select an average treatment applied to an average patient with average outcomes. Yet, it is empirically evident that cancer is a complex evolving system that does follow some rules that are known and can be modeled and predicted mathematically in each patient. For instance, we know that cancer is a proliferative process that outcompetes the otherwise normal tissue to grow. Cancer cells have the ability to engage and co-opt their local environment to their benefit for growth and invasion. While these cancer cells hack normal rules of biology to their advantage, other known or identifiable biological and physical rules drive and/or constrain phenotypes. Based on these processes, the seemingly unwieldy cancer process can be formalized as mathematical equations which can be parametrized for each patient’s data (e.g. imaging, molecular, tissue). Every cancer patient deserves their own individualized equation (TEDx: http://bit.ly/1p1pl8A), a personalized parameterization of their disease evolution that can be exploited to guide and optimize his or her care.

### Current and future challenges

The potential machine learning (ML) or artificial intelligence (AI) applications to healthcare have recently received particular notice in the media with IBM’s Watson, applications to radiology and diagnoses aided by wearable technology amongst many others. Each of these examples are exciting, but the full promise of personalized medicine remains unrealized. While the amount and types of clinical data being generated for each cancer patient is increasing dramatically, there are no holistic approaches or algorithms available that can incorporate all this data to identify the best treatment for each individual patient. There are, obviously, many reasons for this, but a critical challenge is that cancer is a spatially complex, adaptive process and the data being collected, while vast, is quite limited in that it is showing, at best, infrequent snapshots of extremely small regions of the tumor. Ultimately, this means AI and ML models will not be able to be trained on the right data to make reliable predictions.

Mechanistic models can help. Cancer is fundamentally a physical process subject to the same predictable laws of nature studied in physics and chemistry. Of course, it is also a biological, multicellular evolving ecosystem with critical events happening on an enormous range of spatial and temporal scales from improper DNA methylation to the alteration of an organ’s function. These complex interacting components complicate the interpretation of data and experimental planning. While there are many fields of cancer research, mathematical oncology, a field dominated by mechanistic models, is arguably the best equipped to abstract overarching principles and develop a deeper understanding of how the mechanisms driving cancer can be exploited and shut down. To date, however, there are few models that have bridged the scales necessary to fully utilize all the data generated. Thus, these models provide insight, but are not necessarily adapted to assimilate the breadth and depth of the data.

### Advances in mathematics, technology, or data to meet challenges

**Figure 1.**
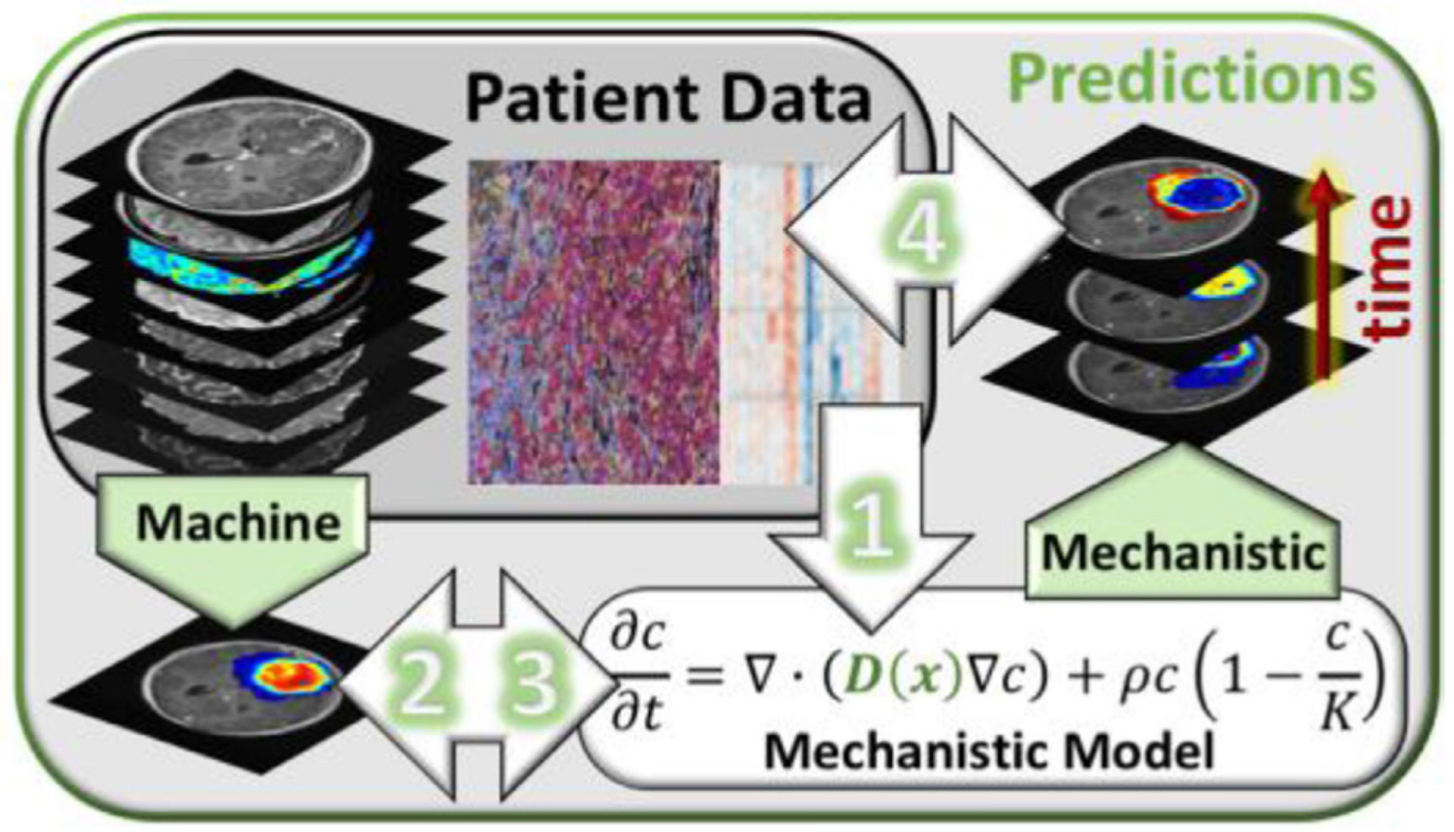
Illustrating the types of advances one might expect to see in the integration of mechanistic modeling into machine learning methods (and vice versa) applied to the case of brain cancer. Mechanistic models, e.g. [1–5], and machine learning, e.g. [6, 7], can interact in multiple ways. (1) ML models can help mechanistic models make sense of multi-scale data to calibrate parameters, e.g. [8, 9]. (2) Mechanistic model predictions can be used as input into ML models to augment spatially or temporally sparse data, e.g. [10] (3) Static outputs from ML models can be used as initial conditions for mechanistic models and (4) ML models and mechanistic models can work together via data assimilation to create spatially and temporally resolved predictions over long periods of time.

These two approaches, insightful mechanistic models and the powerful black box of machine learning, are highly complementary. A grand challenge of our day is to develop methods leveraging both their strengths to create a symbiotic modeling paradigm such that its whole is greater than the sum of its parts—one that can both leverage the vast data at our fingertips (machine learning) but also the knowledge we already have gained from that data (mechanistic models). We envision such mergers can and will take many forms, such as those outlined in the following and illustrated in figure 1.

1. Mechanistic Model Calibration via Machine Learning A current challenge for mechanistic models is the incorporation of the vast amount of ‘omic’ data into parameters. ML approaches could be used to identify gene or expression signatures that best correspond with given mechanistic model parameters utilizing cell cultures and preclinical models. Once initial signatures are found, correlations could be validated with *in vivo* human data.
2. Mechanistic Model Outputs as Inputs to Artificial Intelligence Models A critical limitation for AI models is the relatively limited amount (both spatially and temporally) of data available for training. Mechanistic models can be used to create personalized extrapolations of the provided data for utilization in the AI models. This synthetic data could be used as a regularizer or as fully weighted training data and could enhance the model stability when working with smaller sets of data.
3. Actionize Machine Learning Predictions with Mechanistic Models Static outputs from ML models could be leveraged as initial conditions in dynamic mechanistic models to move the ML prediction forward in time. If, for instance, a ML model could take information from the transcriptome to predict local concentrations of cellular constituents, this information could provide an initial condition for models of cellular interaction allowing the mechanistic model to better incorporate data from many different scales.
4. Data Assimilation for Mechanistic Models utilizing Machine Learning Outputs Building on the previous method, if the appropriate data for the ML model is anticipated to be available for a series of discrete time points, such as MRIs, the ML model output can be used within a data assimilation framework to continuously correct predictions from mechanistic models.

### Concluding remarks

Science and data have always had a strong relationship, but in the last decade or so, the term data science has started taking on a specific meaning related to machine learning and artificial intelligence which ironically leaves behind the notion of the scientific method and hypothesis testing. While there is no doubt that the recent explosion of data cannot be fully exploited without such AI methods, mechanistic models offer a strong complementary, hypothesis driven, approach to synthesizing meaning and strengthening predictions that should not be ignored. This is particularly true in the field of mathematical oncology, where the data is often vast and deep but not representative spatially or temporally. Creating fundamental mergers between these two approaches is a critical step to fully realizing the vision of targeted personalized therapy.

### Acknowledgments

The authors gratefully acknowledge the funding support of this work through the NIH (R01CA164371, R01NS060752, U54CA210180, U54CA143970, U54CA193489, U01CA220378), the James S. McDonnell Foundation and the Ben & Catherine Ivy Foundation.

## 3. Data and model standards

James P Sluka^1,2^, James A Glazier^1,2^ and Paul Macklin^1,*^

^1^ Intelligent Systems Engineering, Indiana University. Bloomington, IN 47408, United States of America

^2^ Biocomplexity Institute, Indiana University. Bloomington, IN 47408, United States of America

* Invited author. macklinp@iu.edu

### Status

Cancer presents a complex series of systems problems involving intracellular dynamics, intercellular interactions, and extracellular biochemical and biophysical processes, embedded in a complex and continually changing spatial context. Although single laboratories can contribute important advances, they cannot individually solve the large-scale problems of cancer biology. As a result, consortia of experimental labs, clinical centers, and computational groups are increasingly pooling their specialized expertise to gain new insights into the complexity of cancer [11]. This pooling requires integration not only of heterogeneous data collected by different groups, but also of scientific hypotheses and deductive observations (knowledge capture), and conceptual, mathematical and computational models. Such integration is much more efficient when data and knowledge representations are standardized.

Difficulty in finding, accessing, interpreting, and reusing data, knowledge, and models hinders collaborative cancer research. A lack of standardized data and knowledge representations, inconsistent metadata (e.g. to describe experimental protocols), technical and financial obstacles, and systemic cultural barriers all discourage sharing [12]. Modern genomics and proteomics demonstrate that widespread adoption of data standards enables faster and more efficient scientific progress.

Many biological research communities are developing standards to annotate and share concepts and data. For example, the microarray and microscopy research communities are developing standards for sharing annotated data such as MGED [13] for micro-arrays, OMERO [14] for microscopy, and the National Cancer Institute’s ‘Common Data Elements for Cancer Research’ [15].

Currently a lack of standards impedes sharing of many types of mathematical models and computer simulations of cancer. While mathematical models can elegantly express data-driven hypotheses, their reuse and combination into larger-scale models requires (currently lacking) standardized representations of equations, model assumptions, and the rationale for parameter estimates. The same problems apply to computer simulations.

The systems biology markup language (*SBML*) community has successfully developed standards to describe dynamic biological network models and to enable their translation into executable computer simulations [16]. The SBML standard not only defines mathematical concepts and syntax, but also allows annotation of model components with biological terms (e.g. naming genes and biological processes). Well-constructed SBML models retain their underlying biological descriptions and associated scientific knowledge. Similar standards for representing multicellular data, knowledge and models are critical for cancer research, but they are presently less developed. The Cell Behavior Ontology [17] and multi cellular data standard (MultiCellDS) [18] are steps in this direction. Standards for general knowledge and hypothesis representation are even less developed.

### Current and future challenges

To maximize the value of mathematical models and computer simulations of cancer to the research community, funding agencies, and society, data, knowledge, models, and simulations should be *FAIR*: findable, accessible, interpretable and reusable [19]. Describing models and data using accepted biological nomenclature and maintaining their links with their underlying biological hypotheses would greatly facilitate finding, accessing and interpreting their domain and biological content, maximizing their value by capturing their embedded scientific knowledge for reuse.

To enable sharing and reuse in future research, we must record experimental, clinical, and simulation data using community-driven standards, drawing upon ontologies that precisely define biological terms and relationships. These data must include metadata such as descriptions of experimental and computational protocols that contextualize data and allow replication [12, 19].

Beyond expressing raw data and models, the community must also develop annotations of biological hypotheses, observations and insights (knowledge). Researchers often communicate this information using qualitative conceptual ‘mental models’ or ‘verbal models’ that represent decades of expert learning.

Machine-readable, searchable representations of conceptual biological knowledge would greatly facilitate sharing [12].

Because sharing computational models is largely limited to sharing source code with little documentation and no biological annotations (or worse, executables with no source code), simulations are often unre-producible [19]. Future computational models (and their parameter sets) must be biologically annotated to facilitate their reuse in more comprehensive multiscale simulations. Biological annotations would also make computational models more accessible via search engines, reducing the need for formal repositories and driving further reuse.

### Advances in mathematics, technology, or data to meet challenges

Enabling FAIR research requires robust annotation schemes for biological, clinical, mathematical, and computational data, including context, biological assumptions, and knowledge gained. Relationships and interactions between biological entities and processes resemble graph structures in SBML network models. However, ‘translating’ imaging data into biological annotations will require machine learning approaches that extend beyond present-day image processing and feature extraction tools [12]. More broadly, tools and utilities must develop alongside standards to make standards-compliant science simple and user-friendly, and to integrate it into existing experimental, mathematical, and computational workflows.

We can learn from the SBML community’s experience to develop similarly robust and FAIR descriptions of mathematical and computational models beyond SBML’s interaction-network concepts. Representing spatial effects is particularly challenging. Projects like CellML [20] and MultiCellDS [18] require continued effort to grow from white papers to widely-adopted standards. Synergies are clearly possible. For example, descriptions of microscopy imaging data and multicellular simulation outputs have significant overlap and should admit a common description language.

We also need to harmonize the numerous data and model standardization efforts across biotech and biological communities. These efforts need to coordinate to ensure that emerging standards are consistent, particularly in the biological description of data, experiments, models, and knowledge. Ideally, harmonized standards should apply to many types of experimental observation (e.g. high throughput microscopy), and generalize from cancer to normal physiology and other diseases.

Standards should be designed so that they support, rather than inhibit, creativity. Tools that make annotation and standards compliance easy are critical to voluntary adoption. Properly implemented and extensible standards serve as a conduit to communicate new ideas and allow better connectivity between models, tools, and data.

Well-implemented standards provide value to individuals in the form of increased access to data, models, and tools, and greater impact via reuse. We must also ensure that standards are straightforward to implement across computing languages and platforms, particularly for scientists who focus on developing conceptual and mathematical models.

### Concluding remarks

Sharing cancer data, models, and knowledge using standardized formats and FAIR principles offers substantial benefits. Stable standards will encourage development of shared software that can import annotated data, design models, execute them as simulations, and analyze their outputs. As technologies such as bioprinting advance, the same tools could enable the direct translation of captured knowledge into living experiments.

Technologies for sharing will help us create automated tools that systematically mine biological literature, databases, and knowledge repositories. Sharing technologies and standardized data are essential if machine vision and other learning approaches are to automate the extraction of observational insights from experimental, clinical, and simulation data [12].

Widespread adoption of standards and adherence to FAIR principles will transform cancer research into an ecosystem of mutually compatible concepts, data, models, and tools. Such standardization will enable community science that exceeds the sum of its parts and accelerates progress in treating cancer.

### Acknowledgments

PM was funded by the Jayne Koskinas Ted Giovanis Foundation for Health and Policy, the Breast Cancer Research Foundation, the National Institutes of Health (NCI U01 CA232137–01, NIH OT2 OD026671–01), and the National Science Foundation (1818187). PM and JAG were funded by National Science Foundation grant 1720625. JPS and JAG were funded by National Institutes of Health grants NIGMS GM111243 and GM122424.

## 4. Multiparametric imaging to enable rigorous tumor forecasting

David A Hormuth II, Angela M Jarrett, Ernesto A B F Lima, J Tinsley Oden, George Biros and Thomas E Yankeelov

Oden Institute for Computational Engineering and Sciences, The University of Texas at Austin, Austin, TX 78712, United States of America

### Status

While mathematical modelling of tumor growth dynamics has a long history, current approaches are limited in their practical applicability. There exist three main reasons for this. First, tumor dynamics are extremely complicated because of the underlying physical and biological processes, as well as the variability across individuals. Second, we cannot easily conduct relevant experiments; we can, for obvious reasons, only observe. Third, the data we do observe is limited; we typically have few measurement points via anatomical imaging or biopsy. Despite those formidable challenges, there is hope. Several imaging methods exist that can provide quantitative information noninvasively, in three dimensions, and at multiple time points. Magnetic resonance imaging techniques can quantitatively characterize vascular properties, cellularity, pH, and pO_2_ [21].

Furthermore, positron emission tomography can quantitatively characterize metabolism, proliferation, hypoxia, and various cell surface receptors [22]. These measurements can be made throughout therapy; thus, imaging allows models to be constrained with patient specific data rather than tabulations from the literature or animal studies.

**Figure 2.**
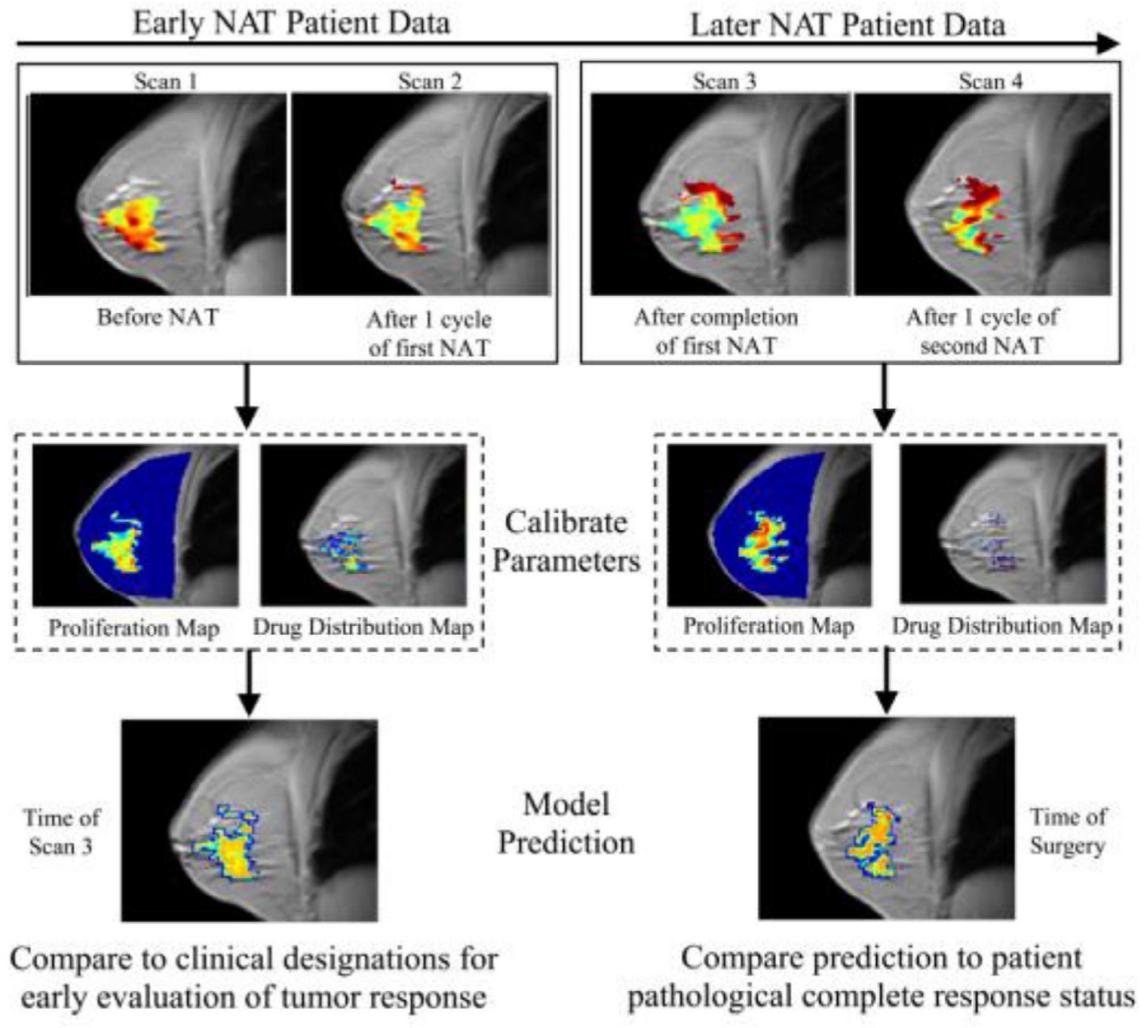
A breast cancer patient was scanned by magnetic resonance imaging at four points during neoadjuvant therapy (NAT). The first two scans (left set of images) are used to calibrate model parameters for predicting response observed at the third time point. The last two scans (right set) are used to update parameters for predicting response observed at the time of surgery.

In recent years, there have been increasingly successful examples of integrating patient-specific information with mechanism-based mathematical models designed to predict the spatio-temporal development of cancer. Successful efforts matching model predictions with clinical observations have been realized in cancers of the breast (figure 2, [23]), kidney [24], and brain [25].

### Current and future challenges

If a mathematical model could faithfully predict the spatiotemporal evolution of an individual’s tumor, then patient-specific hypotheses could be tested *in silico*, thereby allowing the optimizing of intervention for the individual patient using the specific characteristics of their own unique situation. Unfortunately, this vision is quite disconnected from the current state-of-the-art, and remains a grand challenge in mathematical oncology. Currently, the response of solid tumor to therapy is monitored by changes in tumor size as measured by physical exam or anatomically-based, imaging; unfortunately, these methods cannot determine response as anatomical changes are often temporally downstream of underlying physiological, cellular, or molecular changes. Early and accurate predictions would enable replacing an ineffective treatment with an alternative regimen, thereby potentially improving outcomes and curtailing unnecessary toxicities. The development of mechanism-based, predictive mathematical models that could address this fundamental shortcoming in cancer care would represent, without question, an enormous improvement in the human condition. The major challenges to achieving this goal can be summarized by the following three questions:

1. Among the enormous number of models covering a huge range of physical and biological events, which models are the ‘best’ for predicting quantities of interest?
2. How is the uncertainty in the predicted quantities of interest quantified and how can the model predictions significantly improve patient care?
3. How can one access data to inform computational models and, at the same time, cope with experimental noise and errors in the systems used to collect and process data?

### Advances needed to meet the challenges

The importance of accurately predicting eventual patient response is difficult to overstate. The field of numerical weather prediction provides an excellent example of how practical, predictive oncology can be achieved. Weather prediction employs satellites to provide a diagnosis of the state of the atmosphere, which is then evolved forward by meteorological models to provide a prognosis (i.e. a ‘forecast’) of the atmosphere’s future state. Similarly, imaging provides a diagnosis of the state of a cancer, which can then be evolved forward by mathematical tumor models to provide a prognosis of the tumor’s future development [26]. With this analogy in mind, we discuss the advances that must be made to address the three questions of the previous section.

It is imperative that the field constructs mathematical models based on the established principles of physics and cancer [27]. While phenomenological models can provide practical advances for predictive oncology (e.g. the linear quadratic model of radiobiology [28]), they are fundamentally limited in their ability to describe the underlying biology and, therefore, the precise effects of any therapeutic intervention. Unfortunately, this has proved to be a terribly difficult undertaking as we do not yet have the *F* = *ma* of cancer. *In lieu* of this fundamental relation, we have advocated for developing families of models (reminiscent of the approach used in weather modelling), each with its own set of biological and physical assumptions [26, 29]. These models are then calibrated with rationally selected, patient-specific data, before being subjected to a Bayesian methodology that both selects the optimal model and then validates its ability to accurately predict the spatiotemporal development of an individual patient’s tumor.

The sentiment that we are ‘swimming in data’ is often expressed, but it is a tremendous oversimplification. While it is true that there are volumes of clinical data available, it is not of the kind that is readily integrated into mechanism-based models. We may be swimming in data, but we are in the wrong pool. Advances in biomedical imaging are now providing us with the appropriate tools to quantitatively characterize cellular, molecular, and physiological processes that can constrain the next generation of predictive models.

### Concluding remarks

It must be stressed that building data-informed, mechanism-based mathematical models of cancer is a fundamentally different approach than relying only on ‘big data’ [30]. This is not to dispute the fact that statistical inference is of critical importance; but rather, by its very nature it is based on statistical properties of large populations in which conditions that prevail in specific individuals are hard to detect. That is, the ‘big data-only’ approach cannot account for subtle changes in the individual patient—indeed, the very characteristics that make us individuals—over an extended time. It is critical to unite such population-based statistical data with patient-specific measurements and with patient-specific mathematical models that can predict patient-specific changes associated with cancer initiation, progression, and response to therapy. This transformation is inevitable.

### Acknowledgments

NCI R01CA142565, R01CA186193, U01CA174706, CPRIT

## 5. Cancer screening and early detection with modeling

Kit Curtius^1^ and Ibrahim Al Bakir^1,2^

^1^ Centre for Tumour Biology, Barts Cancer Institute, Queen Mary University of London, London EC1M 6BQ, United Kingdom

^2^ Inflammatory Bowel Disease Unit, St. Mark’s Hospital, London HA1 3UJ, United Kingdom

### Status

Cancer screening aims to detect neoplastic changes early for curative intervention. Current programmes, however, suffer from both *overdiagnosis* of benign lesions and *underdiagnosis* of dangerous lesions missed by screening [31]. Consequently, improvement in screening success is an important health policy research area, and one primed for quantitative assessment. In this Roadmap article we argue that mathematical modeling of tumor evolution will underpin radical improvement in the effectiveness of screening and surveillance.

For clarity within a varied literature, the term *cancer screening* refers to initial testing for the presence of a specified neoplastic change of interest in the body (e.g. detection of premalignant or malignant lesions). Subsequent tests that are offered after an initial screening diagnosis are defined as *surveillance screens*. A *biomarker* is a measurable, objective indication of a biological state (e.g. aneuploidy or tumor size) associated with relevant preclinical disease states potentially before symptoms develop.

The length of time between the early detection of a preclinical state and the future clinical detection is called *lead time*, which depends on the nature of the biomarker measured. If the age at completion of lead time surpasses patient lifetime, this patient will be considered an *overdiagnosed* case for that cancer. Lastly, *risk stratification* refers to prognostic subgrouping offered to patient groups based on screen outcome.

**Figure 3.**
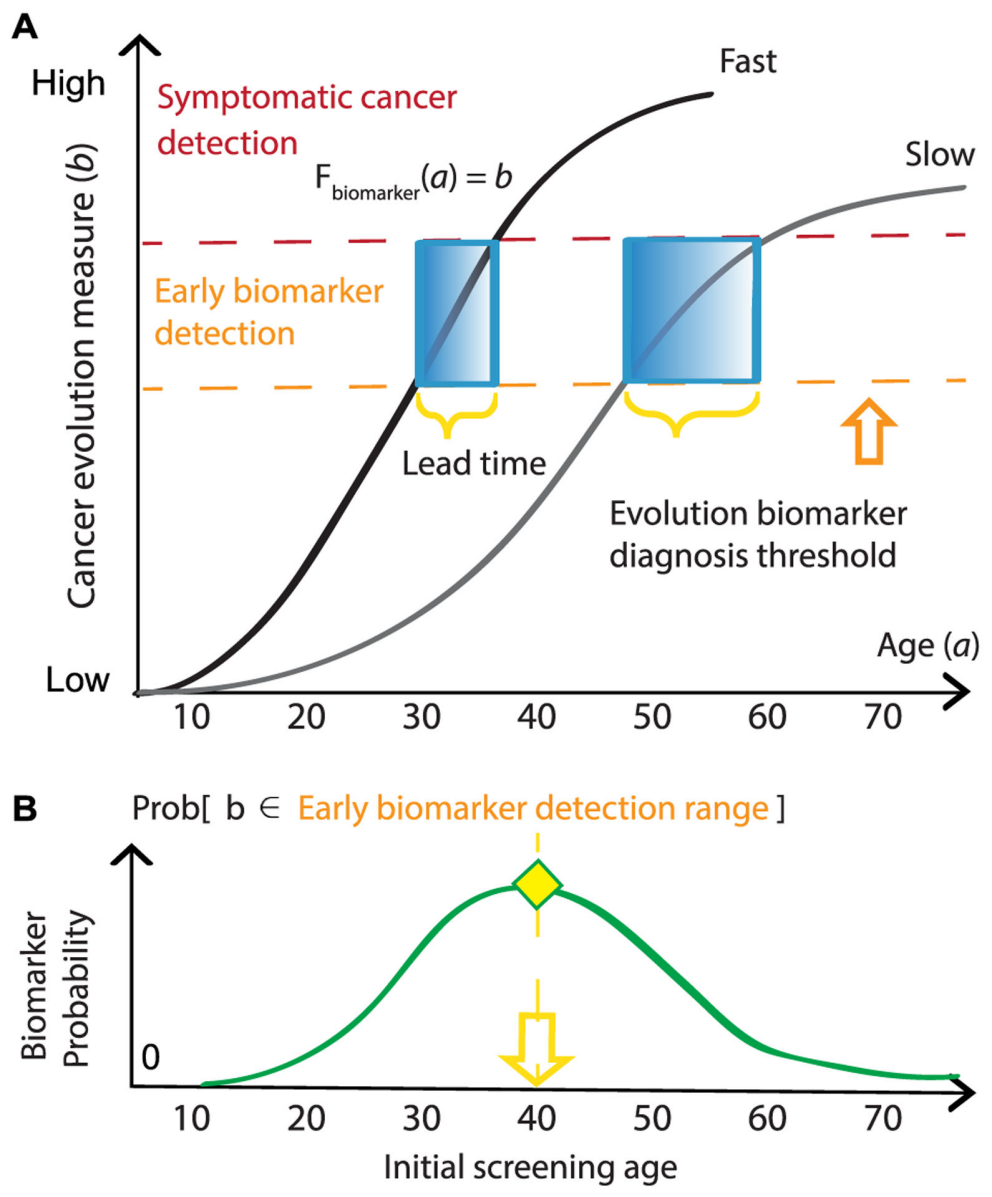
(A) Evolutionary trajectories of slow versus fast carcinogenesis correspond to longer versus shorter lead times for potential clinical intervention, respectively. (B) Screening programme design aims to maximise positive biomarker yield in an average at-risk population.

Currently, screening design uses data from epidemiological studies but does not typically consider tumor evolution, which ultimately determines disease development timescales. From a biological perspective, early detection of biomarkers that alert us to cellular changes along the path to cancer (e.g. premalignant metaplasia detected in biopsy sample histology or circulating tumor DNA present in liquid biopsies) is the clinical manifestation of *field cancerization*, wherein groups of cells have acquired some but not all of the phenotypes necessary for clinical malignancy [32]. If we determine the pattern and pace at which normal cells become cancerized in their microenvironments, we can utilize multiscale data within mathematical models of carcinogenesis to evaluate and predict the efficacy of screening strategies for early detection *in silico* (figure 3).

The impact of this research will be to develop novel methodology capable of (1) utilizing screen data to assay the carcinogenic process *in vivo*, and (2) robustly assessing and refining screening practices using *mechanistic forecasting* to improve early detection and personalize clinical recommendations.

### Current and future challenges

Current screening prevents cancer deaths but there are many areas for improvement. Below we discuss a few main challenges faced.

#### 1. Defining success and introducing bias

To measure the efficacy of screening, investigators may perform randomized control trials (RCT) to compute relative risks of endpoints such as cancer incidence and mortality between screened and unscreened populations [31]. Although intended to reduce biases, these studies are not designed to predict long-term trade-offs in costs versus benefits between alternative screening/lifelong surveillance regimens, which instead require decision modeling [33–39]. The choice of metric for quantifying early detection-associated costs (e.g. decreased patient quality of life or burden to the healthcare system per overdiagnosed case) versus benefits (e.g. life-years gained or cancer precursor eradication per screen) will vary cost-effectiveness results.

#### 2. Choosing an appropriate computational model

Model selection for the established outcome must capture the essential features of disease progression from birth to death. These might include epidemiological features such as patient smoking history [33] and sampling modality such as tissue [34] or blood [35] biopsies. Importantly, the relationship between biomarker level and time (*F*_biomarker_, figure 3) is often sensitive to clinically *unobservable* events, such as metastasis initiation [36] and false positive diagnoses [37], potentially confounding reports from medical exams and contributing to inaccuracy of mathematical formulation.

#### 3. Handling stratification and heterogeneity

Based on biomarkers measured from screens, patients are stratified into ‘low’ and ‘high’ risk groups. Prognostic cut-offs between groups are ultimately arbitrary and can be subject to medical discretion. Two common issues with this practice are that studies rarely consider time-dependent implications (e.g. a high-risk mutated cell may not survive long enough to initiate tumorigenesis) and most rely on a small subset of risk factors measured at a single time point rather than a holistic view of diverse patient background (e.g. family history of cancer, lifestyle, immune system’s innate ability to eliminate mutated cells, adverse mutations). Moreover, the challenge is to accurately characterize the unique evolutionary trajectories of individuals (figure 3(A)), while still recommending useful screening programs that capture average population behavior (figure 3(B)).

#### 4. Testing and performing model validation

New technologies for early detection are rapidly developing, but it remains costly to obtain large, longitudinal cohort follow-up data to robustly assess outcomes and validate screening recommendations in those with an adverse biomarker state; such clinical evidence will be required before altering the existing screening regimens.

### Advances in mathematics, technology, or data to meet challenges

#### 1. Collecting population data for research use

National health institutes are increasing support of early detection studies obtained from prospective cohort and large-scale population screening, which will better inform parameters used in modeling such as disease regression rates and more subjective measures like quality-adjusted life-years used in economic evaluations.

#### 2. Mathematical developments

**Figure 4.**
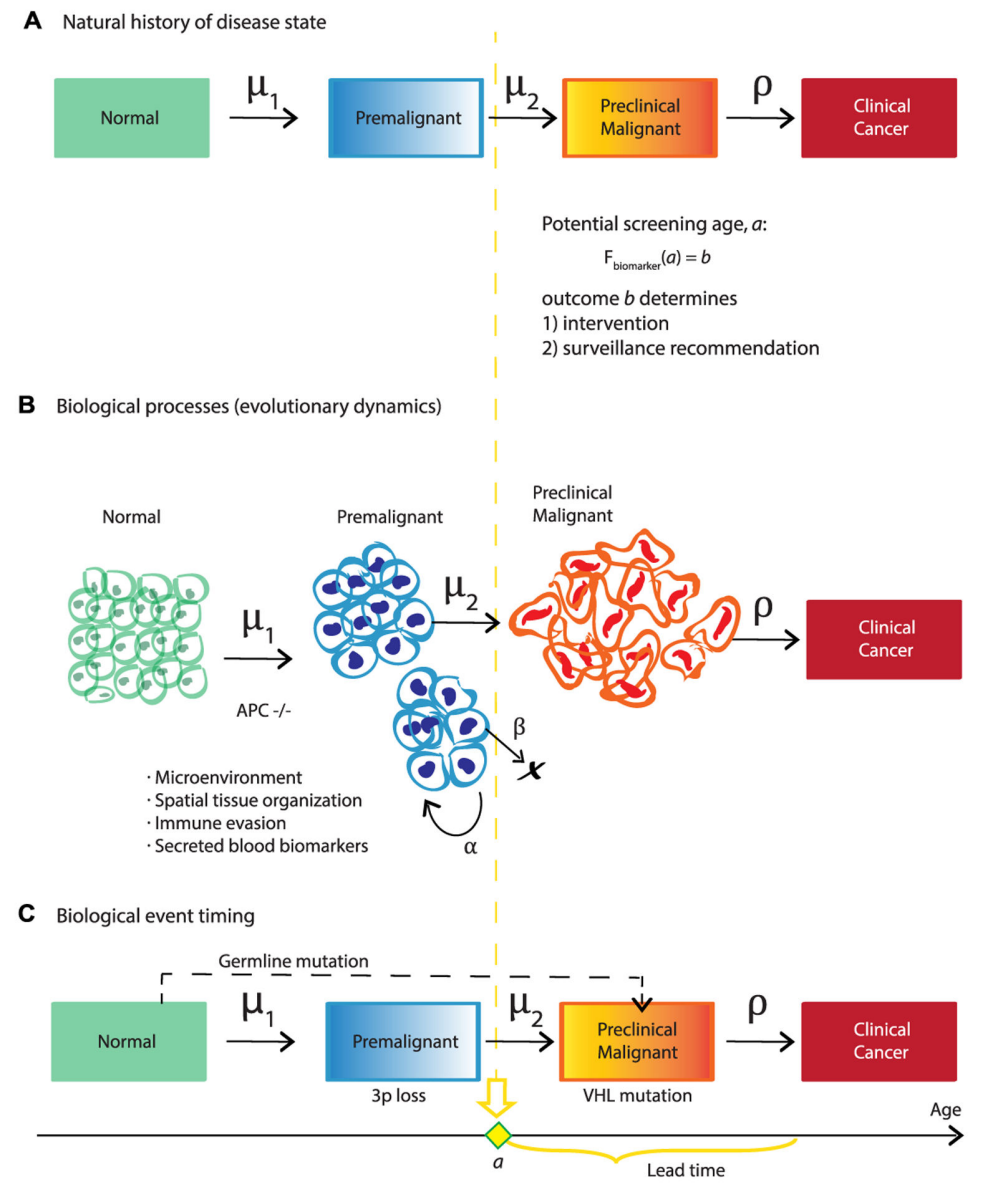
Three models of carcinogenesis to evaluate screening. (A) Natural history models may also explicitly include misdiagnoses into transition rates. (B) Biological models can incorporate growth rates initiated by tumor suppressor gene inactivation (e.g. APC in colorectal adenomas [39]). (C) Inferred biological event models can include alternative pathways such as known germline mutations (e.g. VHL in patients with von Hippel-Lindau disease [40]).

Mathematical modeling (stochastic processes, evolutionary theory, dynamical systems, differential equations) is a framework that can help us to rigorously answer the questions of ‘*when*’ to screen individuals for cancer indications, and ‘*who*’ will benefit most from particular surveillance regimes and clinical intervention. There is a clear need for novel methods to combine models with classical biostatistics commonly used in cancer risk stratification studies for clinical translation. Three current methodologies for assessing cancer screening with modeling are shown in figure 4. These include Markov chains for natural history of disease trans ition [33, 37, 38], biologically-based models that can incorporate evolutionary dynamics like clonal expansions and biomarker shedding in diverse lesions [33–36, 39], and biological event timing models that infer critical genetic events during carcinogenesis [40]. Moreover, biologically-based models could inform the transition probabilities of the Markov approach. The aim of all these models is to quantify long latency periods of premalig-nancy on a patient’s forecasted evolutionary trajectory. These periods provide a window for therapeutic intervention when detected during effectively-timed screens.

#### 3. Modern technologies for sensitive and specific early detection biomarkers

Rapid advancements in multi-omic and optical imaging technologies allow for the diagnoses of precancerous and early cancer lesions at higher resolution and at decreasing cost to the healthcare system. These will provide researchers with better understanding of patient-specific disease evolution, and ultimately result in personalized prevention efforts becoming a clinical reality. Taking a holistic view and studying disease evolution at adequate power will require huge amounts of well-annotated patient data, but with digitization of medical records and large population cohorts currently undergoing follow-up, we envisage this may be feasible within the next 30 years.

#### 4. Performing virtual trials in silico for rigorous model selection and testing

Well-calibrated mathematical models provide a cost-effective, ethical means for simulating virtual cohorts of patient outcomes to judge the effectiveness of a screening/surveillance regime both across a population and in individuals. Bayesian approaches and deep learning of large clinical datasets will also enhance statistical inference of unobserved events that drive carcinogenesis timing; such modeling will be necessary in future early detection research as it is not technically feasible to measure many aspects of tumorigenesis (such as single progenitor cell initiation) in the patient cohort itself. Moreover, this dynamic, computational approach is a straightforward method to continuously test and recommend modifications to screening/surveillance guidelines (e.g. to reflect subsequent technological advances in endoscopic optical imaging), as opposed to the current situation wherein such guidelines are updated on average once per decade.

### Concluding remarks

There is exciting potential for mathematical modeling in addressing the challenges of cancer early detection, alongside developments in biomarker discovery and validation. Modeling cancer screening will allow researchers to examine the underlying cause of the vast inter- and intra-patient heterogeneity we currently observe clinically during disease progression in a robust and unbiased way. It will be possible to create explicit formulations for the dynamics of biomarker changes in the body and to formulate quantitative functions for screening efficiency in order to optimize cancer screening and surveillance scheduling.

In reality, all cancers form from a series of evolutionary changes that may be detectable (and potentially preventable) if we *anticipate* and *seek* such changes during screening, and track them during surveillance to direct clinical action. In our increasingly integrated world, patients, doctors, policy-makers, and mathematical modelers will be required to engage in interdisciplinary science efforts to best answer questions about how to beat cancer early.

### Acknowledgments

This work was supported by UKRI/Rutherford Fund Fellowship (KC), the Medical Research Council (MR/P00122X/1 to IAB) and the St Mark’s Hospital Foundation Research Grant (RES198 to IAB).

## 6. Cancer dynamics

Dominik Wodarz^1^ and Natalia Komarova^2^

^1^ Department of Ecology and Evolutionary Biology, University of California Irvine CA 92697, United States of America

^2^ Department of Mathematics, University of California Irvine, Irvine, CA 92697, United States of America

### Status

The development and progression of cancer is driven in part by evolutionary processes within the underlying tissue cell populations. Cells that are subject to homeostatic regulation in healthy tissue acquire mutations that can alter the properties of the affected cell. Many such mutations can lead to a selective disadvantage while others do not change the fitness of the cell or confer a selective advantage. Accumulation of one or more such driver (advantageous) mutations can allow the cells to escape homeostatic regulation and to proliferate out of control. These clonally expanding cells can in turn accumulate further mutations that result in increases in heterogeneity in the cell population and in further progression of the tumor. Such evolutionary processes are not only crucial for the disease development, but can also contribute to resistance against cancer therapies. It is therefore crucial to gain understanding of both the evolutionary principles according to which tumors progress, and the mechanisms by which treatment resistance evolves.

Mathematical models form an integral part in the analysis of evolutionary dynamics in general, and the same applies to evolutionary dynamics in the context of tumors [41, 42]. Mathematical and computational work has contributed insights both into aspects of tumor initiation and progression, and into the principles of resistance evolution [43]. Important measures that have been investigated include the probability that mutants resistant against a given treatment regime exist in the tumor cell population at the time when treatment is started; the expected number of mutants that are present at the time when treatment is started; the probability that mutants with certain characteristics become fixed in healthy tissue or an emerging tumor; the time it takes for mutants to rise towards a certain threshold level, etc. In the context of specific tumors, it has been possible to measure some of the main parameters underlying such models for individual patients. One example is chronic lymphocytic leukemia [44]. Division and death rates have been measured by administering deuterated water to patients, radiological imaging has been used to estimate the total tissue tumor burden, and model fitting to clinical data has been used to estimate kinetic parameters underlying treatment responses. With the knowledge of such patient-specific parameters, the mathematical modeling approaches can in principle be used to make individualized predictions about treatment outcomes, such as the time to resistance-induced relapse against targeted therapies [45]. They can further be used to explore alternative treatment options with the aim to prolong the duration of tumor control.

### Current and future challenges

**Figure 5.**
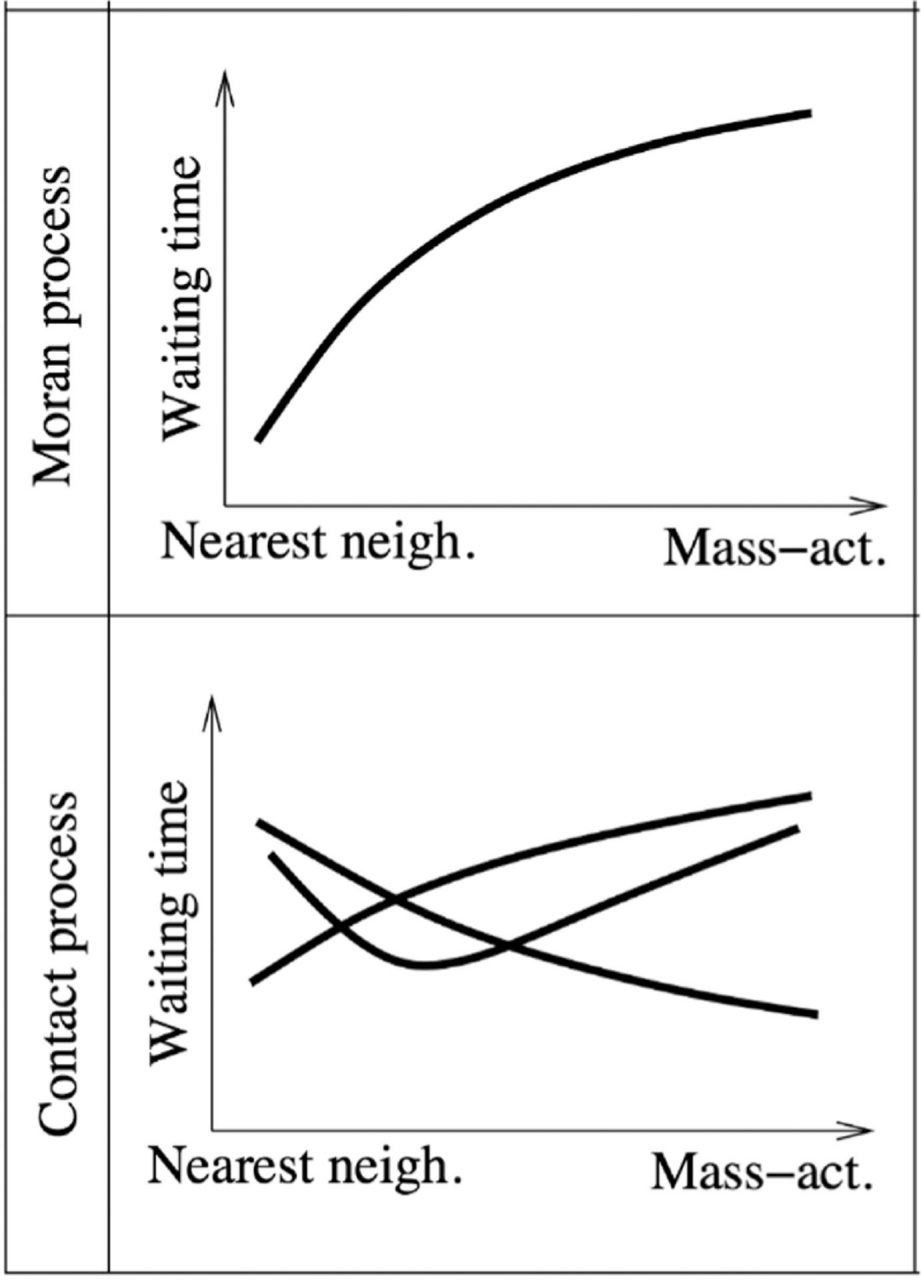
Schematic illustrating the different effects spatial restriction can have on the waiting time until a fitness valley is crossed, in the Moran Process and the contact process. Nearest neighbor interactions represent the strictest degrees of spatial restriction, while mass action corresponds to perfect mixing of cells.

Much of the work described so far has been performed under the assumption that there is no spatial structure in the cell population, i.e. that cells mix well with each other. This might be a reasonable approximation for some leukemias, but is an unrealistic assumption for solid tumors, which are characterized by complex spatial structures. A variety of spatial computational models of tumors have been developed to study different questions, e.g. [46], notably mechanistic models of tumor growth and vascularization have been successful. Many aspects of the evolutionary dynamics of mutant populations in spatial settings, however, remain poorly understood. Interestingly, analyses of spatial evolutionary processes performed so far indicate that the dynamics can be significantly affected by spatial structures, often in complex ways. An example is the process of fitness valley crossing, where an advantageous phenotype requires the accumulation of two (or more) separate mutations, each of which is individually deleterious or neutral. Such evolutionary pathways have been documented to occur in the context of many cancers. An example is the inactivation of tumor suppressor genes, such as the APC gene in colorectal cancer, where both copies of the gene must lose function for the cell to become advantageous. It turns out that the evolutionary timing of fitness valley crossing depends on the exact assumptions on the spatial dynamics [47] (figure 5).

In the spatial Moran process model that assumes constant cell populations, spatial interactions were found to accelerate the rate of fitness valley crossing. By contrast, in contact processes that do not assume constant cell populations, the rate of fitness valley crossing could be accelerated or delayed, and there could even be an optimal degree of mixing that maximizes the rate of evolution.

Studies of single mutant dynamics in spatially structured cell populations have also shown that basic mutant dynamics in space are different compared to well-mixed scenarios [48]. The fate of mutants can depend on the timing and the spatial location of mutant emergence. Mutants that are generated relatively early and at the surface of an expanding spatial cluster of cells can grow to relatively large numbers (also referred to as ‘jackpot’ mutations). On the other hand, mutants can become surrounded and encased by wild-type cells, which limits their growth and introduces and element of competition between mutant and wild-type cells, even though the tumor mass is characterized by unbounded growth. A better understanding of how evolutionary processes contribute to cancer development in such settings is crucial for improving therapies. It is especially important is to gain understanding of how mutants clones defined by different susceptibilities to specific therapies develop in such spatial scenarios, both in the presence and in the absence of treatment.

### Advances in mathematics, technology, or data to meet challenges

As more information is obtained about the spatial evolutionary dynamics of tumors, both experimentally and theoretically, spatial computational models will increasingly form the basis for simulating disease progression and therapy outcome for specific scenarios and individual patients. In contrast to investigating basic principles of evolutionary dynamics, however, these applied questions will require the simulation of tumor growth and evolution at realistically large population sizes. Because this brings with it significant computational costs, the simulation of cell populations that reach sizes between 10^10^ and 10^13^ cells becomes unfeasible. The problem lies in the fact that while the overall tumor population size is very large, mutant cell populations exist initially at very small numbers, which requires stochastic simulations. The time step in stochastic simulation algorithms decreases as the overall population becomes large, thus rendering such computer simulations impractically slow. One way in which this problem has been dealt with is to assume smaller cell populations and higher mutation rates, hypothesizing that the dynamics scale in realistic ways. It is, however, currently unclear whether this holds true. Therefore, to be able to simulate and predict tumor development and treatments at realistically large population sizes and at realistically small mutation rates, novel computational methodologies are required. To this end, a modeling approach has been proposed which assumes that the tumor consists of discrete microlesions; cells can migrate from microlesions to establish new ones, which can all grow over time, and new driver mutations can be generated [49]. From this model, the average behavior can be obtained analytically, which allows simulation of tumor dynamics and evolution at large sizes. In order to capture the stochastic dynamics of various mutant types, however, methodologies need to be developed that allow the stochastic description of small mutant clones in a spatial setting in realistic time frames. Deterministic partial differential equation approximations of such spatial, stochastic processes generally do not yield accurate time series. In the context of mixed populations (where stochastic dynamics are simulated e.g. with Gillespie’s method), novel computational approaches have been developed (e.g. the Next Reaction Method and Tau-Leaping methods), which try to address these difficulties. There is also an important push in the development of hybrid stochastic-deterministic approaches, where small populations are handled stochastically, while larger populations are described deterministically. Such approaches have been typically employed in the field of physical chemistry but have not significantly penetrated the studies of population dynamics and evolution, presumably because they can rely on theoretical concepts (e.g. Langevin’s equation), which are not very common in these fields. At the same time, such approaches would be very useful for the field of mathematical oncology, as demonstrated by a recent study [50]. Application of such methodology to spatial dynamics, however, is a complicated extension, the development of which will be as challenging as it is important. The ability to simulate spatial tumor evolution at realistic population sizes and mutation rates will be central to the development of clinically applicable computational models of tumor evolution, which can be used for the personalization of therapy regimes.

### Concluding remarks

The importance of spatial genetic heterogeneity in tumors has penetrated clinical and experimental cancer research. In various cancers, data indicate that a tumor mass can consist of regions that are genetically distinct and that contain different mutants that can influence the susceptibility of these cell clones/spatial regions to therapies. The emerging biological details about evolutionary patterns in spatially structured tumors will allow appropriate computational models to make more accurate and clinically relevant predictions regarding disease course and treatment outcome, and the availability of efficient computational methodologies will be of central importance in this respect.

## 7. A single-cell topological view of cancer heterogeneity and evolution

Luis Aparicio*, Mykola Bordyuh* and Raul Rabadan

Department of Systems Biology, Columbia University

* These authors contributed equally to this work.

### Status

A tumour is a dynamic disease of the cell that, through alterations in its genome and epigenome, leads to its uncontrolled proliferation. Tumours are found to vary dramatically across patients (inter-tumour heterogeneity) and across cells within a tumour (intratumour heterogeneity). Heterogeneity has been found to be a major factor in cell adaptation driving spread and response to therapy [51].

With the advent of high throughput sequencing [52], there has been a dramatic development in the characterization of inter-tumour diversity. Large scale efforts, like The Cancer Genome Atlas or the International Cancer Genome Consortium, have portraited the molecular make up of thousands of tumours generating diverse large-scale biological datasets. The need to extract useful biological and clinical knowledge from these efforts have highlighted the necessity for new mathematical and computational methods to analyse and integrate them.

The past few years have witnessed the further development of a variety of techniques enabling single-cell molecular measurements, including sequencing the DNA of single cells, or measuring their mRNA, methylation, chromatin state or protein levels [53, 54]. Single-cell RNA sequencing constitutes a powerful technology to address the problem of intra-tumor heterogeneity, enabling the quantification of transcriptome landscapes at single-cell resolution, and providing a tool to observe the dynamics of tumor evolution.

However, single-cell sequencing data comes with some unique analytical challenges. These challenges can be appreciated in dynamic biological phenomena like cell differentiation or tumor evolution, continuous processes where traditional clustering methods may not be suitable. While clustering tries to split data into seemingly distinct sets, the analysis of dynamic processes needs methods that can capture the continuous relation between cellular states. Topology is a branch of mathematics that studies continuous transformations of geometrical objects. Topological data analysis (TDA) adapts techniques of topology to extract information from the geometric and topological data structure. This makes TDA amenable to deal with continuous data structures and therefore, to analyze single-cell data of dynamic biological processes, including cancers.

### Current and future challenges

Ambitious large-scale single-cell projects aim to provide atlases of millions of cells pushing the analysis into the paradigmatic ‘Big-Data’, high-dimensional scenario. The variety of single-cell platforms and associated unique technological challenges bring an additional layer of complexity into the analysis. Associated technological problems vary across platforms and include drop-out effects, big sparsity of the data (on the order of 90% of the inputs) and noisy biological or technological variability in gene expression (typically, around 99% of the variability is associated with the noise). On the other hand, the discovery of rare subpopulations and transitional cell states, which may amount only to a dozen per experiment, present unique computational challenges. Here, we would like to emphasize two mathematical properties of the underlying processes, that are useful when analyzing single-cell data [55]: the **continuity**, associated with cell differentiation and the **locality** property, important in identification of different branches of cell differentiation and small subpopulations.

**Figure 6.**
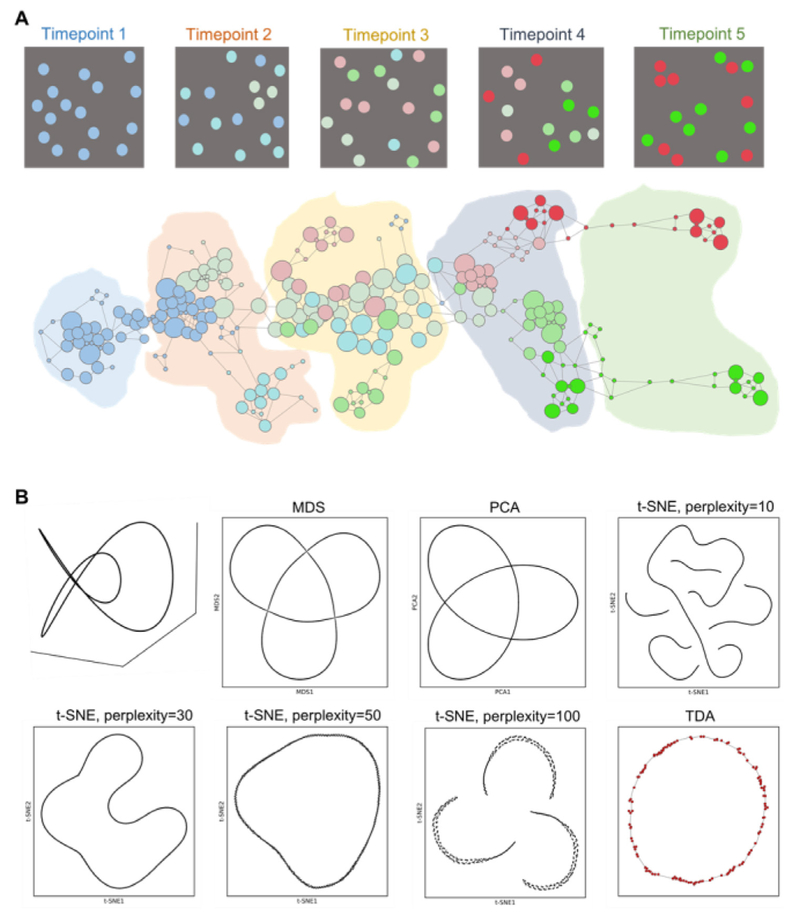
(A) Simulation of a longitudinal single-cell analysis with datasets at different timepoints. Different colours represent different cell types or states. In the down side, a TDA representation. (B) Comparison of TDA and traditional algorithms for dimensional reduction, as multidimensional scaling (MDS), principal component analysis (PCA) and t-distributed stochastic neighbor embedding (t-SNE).

In figure 6, we highlight these two important attributes and principles. In figure 6(A), we show the schematics of cell differentiation at different time-points. Traditional clustering techniques only provide limited information about well-defined cell states, failing to explore continuous nature of cell differentiation processes. Topological representation of the processes, depicted in figure 6(A)(bottom), captures both continuous structure of the process and well-defined states of cells, associated with clusters. In figure 6(B), we illustrate the locality property, important in preserving differences in cell populations, that otherwise are disregarded in lower dimensions. If locality is not preserved, close cell types in high-dimensional spaces, but biologically distinct can be artifactually misrepresented as close (even identical) points in the reduced space. As an example (see figure 6(B)), we took a 3D ‘Trefoil’ curve, where every point is distinct in the original space. Low-dimensional representations, as MDS, PCA and t-SNE algorithm among many others, tend to create artifacts, by breaking the continuity or failing to separate distinct points. Topological representation respects both continuity and locality of every point.

### Advances in mathematics, technology, or data to meet challenges

**Figure 7.**
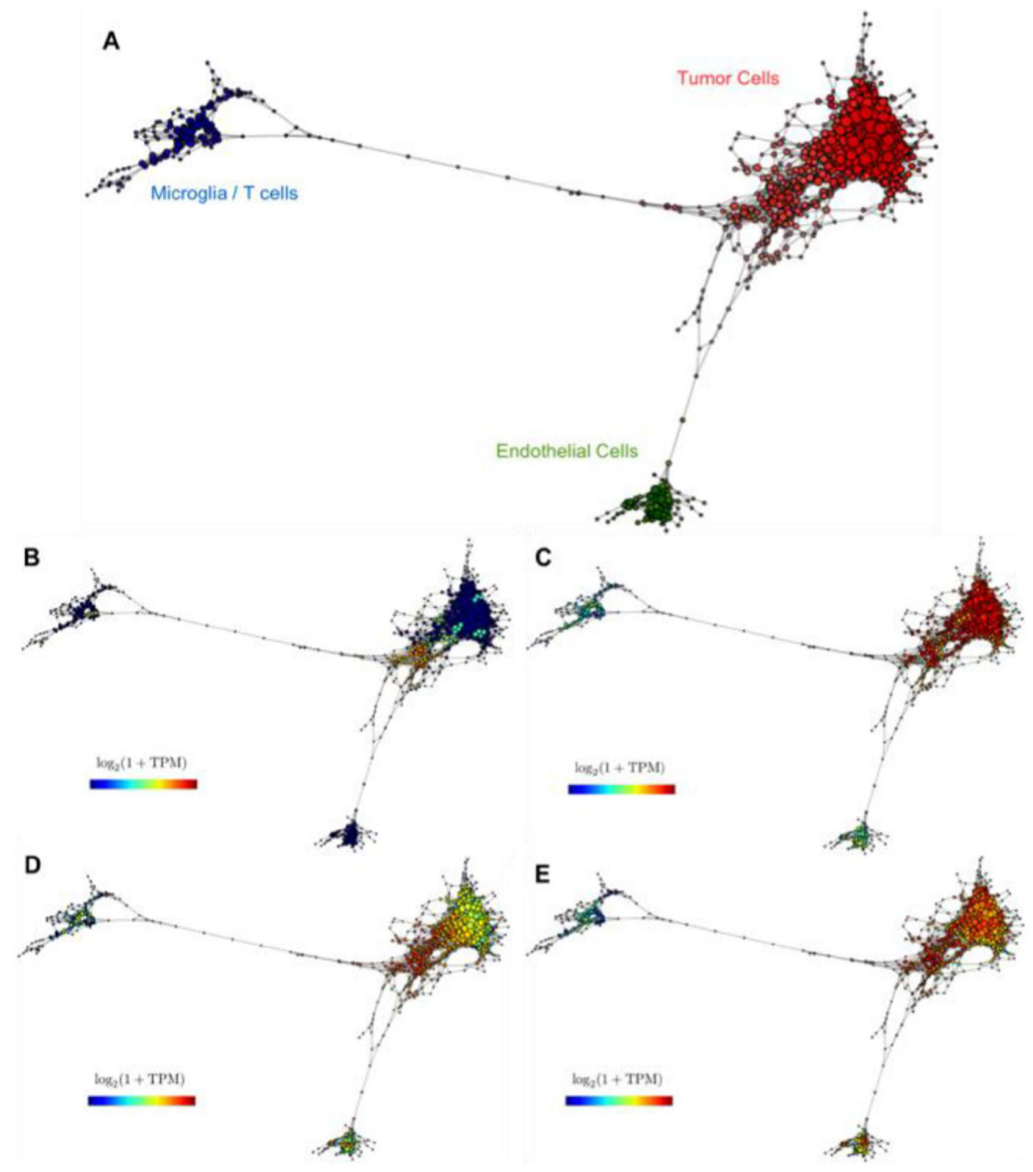
(A) A topological representation of a glioblastoma RNAseq single-cell dataset shows diverse stromal/tumour populations. The expression of specific genes shows similarity with known cell populations: (B) representation of MKI67 expression, (C) oligodendrocyte genes expression, (D) neural progenitor expression, and (E) astrocyte genes expression.

In the past decade, TDA has emerged as a new discipline at the interface between machine learning and algebraic topology. The goal of TDA is to extract and represent information about the shape of data. One can think of single-cell data as points (cells) in a high-dimensional space, where the dimensions correspond to the number of features (typically genes). Most constructions of TDA consist of replacing the original space by a mathematical object called *simplicial complex*, that captures topological features. Simplicial complexes can be seen as generalizations of networks (see figures 6(A) and 7).

One of these properties is the *skeleton* of the space or Reeb space which informs us about the number of connected components in the space or how many holes of different dimensions exist. Mapper [56] is an algorithm based on TDA which constructs simplicial complexes as approximations to Reeb spaces. The result, when applied to single-cell data, is a network where nodes represent sets of cells with similar global transcriptional profiles, and edges connect nodes that have at least one cell in common. In figure 7, we show an example of a topological representation for single-cell RNA sequencing of glioblastoma corresponding to a patient sequenced in [57]. In this case, TDA is not only able to disentangle different tumor and stromal cell populations (figure 7(A)), but also to capture intra-tumor heterogeneity. Figures 7(C) and (E) show a different distribution of astrocytes and oligodendrocytes within the tumor population. Interestingly, from panels B and D one can extract a certain correlation between the neural progenitor signature and the more proliferative cells and more astrocyte-like markers.

### Concluding remarks

Single-cell technologies are a powerful tool to study fundamental aspects of cancer biology at an unprecedented resolution. This is generating an increasing explosion of molecular data and consequently, the necessity of new mathematical methods to analyse it. On the other hand, the intrinsic features of single-cell datasets constitute a challenge for traditional methods of analysis based on combinatorics and clustering. TDA is a modern mathematical set of tools which has a potential to predict the dynamics of intracellular. Remarkably, algorithms based on TDA preserve locality and can capture the continuous nature of the biological phenomena that are analysed at a single-cell level [58–60]. This is crucial to understand better tumour progression and evolution. Future work applying TDA techniques may shed light on key questions in cancer studies like the structure and information contained in the tumour heterogeneity.

### Acknowledgments

The authors would like to thank NIH for their support through the grants: U54-CA193313, U54-CA20997, R01-CA185486, R01-CA179044.

## 8. Metabolism in cancer progression

Stacey D Finley

Department of Biomedical Engineering, University of Southern California, Los Angeles, CA 90089, United States of America

### Status

Altered metabolism is a hallmark of cancer that enables cancer cells to meet the high energetic burden required to support their increased proliferation. Such metabolic reprogramming mediates cancer progression, influences treatment efficacy, and contributes to drug resistance. Thus, it is imperative to better understand tumor metabolism, including metabolic networks in cancer cells specifically, and in other cells that comprise the tumor microenvironment.

*Systems biology* approaches, including computational modeling, are needed to obtain a global understanding of the interconnected metabolites, enzymes, and regulatory mechanisms that characterize cellular metabolism. Systems biology methods allow controlled exploration of the roles of multiple cell types, molecular species, and biochemical reactions in cellular metabolism. Such approaches focus on how individual components of biological systems contribute to system function and behavior, facilitate a deeper understanding of complex biological processes, and provide opportunities to develop new hypotheses and interventions.

There is a substantial and productive history of applying computational modeling to study cancer, from initiation through metastasis [61]. This work demonstrates that computational models, refined by experimental results can reveal effective treatment strategies and provide unexpected predictive insights. In fact, systems biology modeling complements pre-clinical and clinical studies of tumor metabolism. Specifically, systems biology models of cancer metabolism [62] provide quantitative insight into the dynamics of metabolic pathways, are useful in investigating the metabolic mechanisms driving the cellular phenotype and have helped identify potential therapeutic strategies. Thus, systems biology approaches provide new insights into metabolism and can lead to novel therapeutic strategies. When constructed and validated using experimental measurements, systems biology models can be used to perform *in silico* experiments to predict the effects of perturbing the metabolic network. In this way, the models are a valuable alternative to wet experiments that can be expensive and time-consuming.

### Current and future challenges

Many published metabolic modeling techniques have focused on *constraint-based approaches* in which certain physical, chemical, or biological constraints are applied to predict the metabolic phenotypes. These are time-invariant stoichiometric models that predict reaction fluxes, which remain difficult to measure experimentally at the systems-level. Genome-scale metabolic models have been constructed to explore the interconnected metabolic pathways documented to occur in an organism, including cancer-specific models [63]. Such models provide insight into how particular oncogenes influence metabolism, and they help identify specific drug targets and biomarkers. However, constraint-based models are static and fail to capture the kinetic aspects in the system or time-varying heterogeneities that arise due to environmental fluctuations. Additionally, ongoing work is aimed at integrating high-throughput omics data into constraint-based models for a more comprehensive view of the metabolic landscape. Overall, constraint-based models are widely used, and they contribute to our understanding of the role of metabolism in cancer progression.

*Kinetic modeling* is an alternative to constraint-based modeling. When considering processes that are inherently transient, such as the effects of reprogramming of cancer metabolism, kinetic modeling is required to understand the dynamic relationships between metabolic fluxes, metabolite concentrations, and microenviron-mental conditions. Therefore, models that represent the metabolic pathways using a system of nonlinear ordinary differential equations are useful. These kinetic models provide a mechanistic description of the transient dynamics of the system and have been used to identify key enzymes associated with tumor growth and malignancy and predicted the effects of targeting those enzymes [64].

Though highly valuable, kinetic modeling also has some drawbacks. One limitation is that these models require many kinetic parameters in order to accurately characterize the reaction rates. This can be overcome by fitting the model to quantitative experimental data and estimating the parameter values needed to best fit the data. Another limitation is that while these models predict the dynamics of intracellular processes, they rarely account for downstream effects that occur at the cellular and tissue level.

Indeed, multi-scale modeling is a challenge impeding the successful application of systems biology approaches to address clinically relevant questions related to cancer metabolism. There are two aspects to this challenge. The first is a need for robust computational tools to link mechanistically detailed, dynamic models of intracellular metabolism to tumor growth. Second, there is a need for multi-scale models that link a detailed metabolic network model to cell proliferation/apoptosis and account for the heterogeneous, multicellular tumor microenvironment. It is well established that the internal dynamics of metabolism directly influence cancer progression. In addition tumor-stromal interactions play an important role in drug resistance. However, there is a lack of spatiotemporal models that address these critical aspects of cancer metabolism.

### Advances in science and technology to meet challenges

The key to advancing systems biology models of cancer metabolism is to take advantage of existing computational tools for performing multi-cellular simulations and link them with detailed models of intracellular metabolism with cell- and tissue-level dynamics. There are many computational models of cancer cell growth and progression, but few simulate how the dynamics of intracellular metabolism drives tumor growth. Our recent work links a detailed kinetic model of intracellular metabolism to population-level cancer cell proliferation [64] but does not simulate the dynamics of individual cells. Ghadiri and coworkers integrate a constraint-based model with an agent-based model of tumor [65]; however, this model does not evaluate the metabolic fluxes within each cell as time progresses.

Some computational models of cancer predict metabolic interactions between tumor cells. In one example, Robertson-Tessi *et al* incorporate a simplified metabolic model with angiogenesis and tumor growth and predict treatment outcome [66]. They developed a hybrid continuum/agent-based model in which glucose and oxygen are metabolized inside of the cell and directly influence cell growth. However, these models rarely account for the interactions and dependencies between cancer cells and other cells in the tumor microenvironment. Moreover, these models lump together several metabolic reactions and thus cannot predict how targeting specific metabolic enzymes influences cell growth.

Overall, there is a clear gap in the application of multi-cellular modeling combined with mechanistically detailed models, particularly in the context of cancer metabolism. Some tools exist that enable computationally intensive simulations of multi-cellular environments; however, future work is needed to combine these tools with computational models of metabolism reaction networks. We highlight two particular tools: *CompuCell3D* and *PhysiCell*.

- *CompuCell3D* employs lattice-based Glazier– Graner–Hogeweg (GGH) stochastic modeling of generalized cells to simulate tissue-scale behavior [67]. The generalized cell’s behavior (such as proliferation, an increase in volume, migration, and cell–cell adhesion) is driven by its effective energy. The probability that a behavior is performed depends on how that behavior changes the cell’s effective energy (i.e. whether the potential behaviors increase or decrease the energy). Behaviors that lower the cell’s effective energy are preferred. CompuCell3D has been applied in many instances, including incorporating intracellular signaling dynamics that influence the cells’ behavior [68].
- *PhysiCell* implements off-lattice cell agents to model multicellular systems within a biochemical microenvironment [69]. Cell agents interact via direct physical contact or by exchanging diffusible biochemical signals. This tool has been applied to model up to 10^6^ cells in tissue volumes of ~10 mm^3^. In addition, PhysiCell makes it possible to link intracellular networks with cell behavior. For example, a Boolean model of cell signaling has been embedded within each cell to simulate the effects of breast cancer treatment [70].

### Concluding remarks

With multi-scale, multi-cellular models in hand, it would be possible to predict the effects of molecularly targeted metabolism-based therapies on cancer cells, neighboring cells in the tumor, and overall growth of the tumor tissue. Such multi-scale models that include detailed metabolic reactions in combination with cell–cell interactions can be used to identify novel cancer treatment strategies, serving as a framework to hypothesize optimal drug combinations and treatment protocols. We can draw upon work that successfully integrates models of intracellular signaling models with the cell-level response. And in the future, multi-scale models that incorporate both signaling and metabolism networks can even be combined. In conclusion, detailed modeling of cellular metabolism is a clinically relevant application of systems biology modeling that has the potential to significantly impact cancer treatment.

### Acknowledgments

The author acknowledges members of the Computational Systems Biology Laboratory at USC, Dr Paul Macklin, and Dr Shannon Mumenthaler for many inspiring discussions.

## 9. Modeling radiation therapy

Heiko Enderling^1^, Jimmy Caudell^2^ and Eduardo Moros^2,3^

^1^ H. Lee Moffitt Cancer Center & Research Institute, Department of Integrated Mathematical Oncology, FL 33647, United States of America

^2^ H. Lee Moffitt Cancer Center & Research Institute, Department of Radiation Oncology, H. Lee Moffitt Cancer Center & Research Institute, Tampa, FL 33647, United States of America

^3^ H. Lee Moffitt Cancer Center & Research Institute, Department of Cancer Physiology, Tampa, FL 33647, United States of America

### Status

Radiation therapy (RT) is the single most commonly used cancer treatment. More than 50% of patients receive radiation at some point in their cancer care, either as curative monotherapy, in combination with surgery, chemotherapy, or immunotherapy, or as palliative therapy. Current RT practice is based on maximum tolerated dose (MTD) concepts independent of patient-specific biology. Treatment protocols have been derived from average outcomes of long-term empirical practices or large clinical trials and continue because they have produced reasonable outcomes. Despite a long history of medical physics and physical concepts centered around radiation dose delivery technology and safety, few inroads have been made to synergize quantitative approaches with radiation biology and radiation oncology methodologies to optimize RT and treatment personalization. Integrating mathematical modelling with radiation oncology may have an immediate impact for a large number of patients, and help revolutionize how we conceive of and clinically prescribe radiotherapy in the precision medicine era.

### Current and future challenges

RT is the most successful treatment in cancer care that can be given with the intent to cure, as palliative therapy, or potentially with the intent to convert the tumor into an *in situ* vaccine [71]. Whilst a wealth of radiation biology data has been, and continues to be collected, few biological concepts have impacted clinical radiation oncology relative to physical conformality of dose. Historically, dose-escalation trials have focused on increasing log cell kill with acceptable toxicities to provide as much loco-regional control as possible. In current RT practice, the treatment protocol parameters (total dose and dose fractionation) are prescribed *a priori* based on tumor type, disease stage, nodal status, and metastatic burden [72]. Whilst cancer is reminiscent of a complex dynamic adaptive system that may be best understood by perturbing it, to date no concerted efforts have focused on collecting and evaluating longitudinal tumor states to personalize RT, and on identifying markers for treatment adaptation. To fully embrace the clinical potential of RT for the patient population as a whole—and individual patients in particular—we need to (i) determine the optimal total dose to control an individual patient’s tumors, (ii) identify optimal dose fractionation, (iii) explore the synergy of radiation with the patient’s immune system as well as with (iv) surgery, biological agents or chemotherapeutics. To prospectively determine individual treatment protocols, we must be positioned to reliably predict patient-specific treatment responses.

### Advances in science and technology to meet challenges

Quantitative approaches have shown great promise in retrospective analyses of radiation outcomes [73], correlation of pre-treatment tumor growth dynamics with radiation sensitivity [1], and optimization of dose fractionation in pre-clinical models [74]. To fully harness the potential benefits of integrated mathematical oncology, a close dialog between both mathematical and radiation oncology needs to be fostered [72]. With few high-resolution measurements on the cellular and sub-cellular level, hope lies in the anticipated collection of longitudinal data to inform differential equation and equation models to help simulate tumor growth and RT response dynamics.

In a preclinical model of glioblastoma an integrated, iterative approach of experimental data informing a mathematical model, the model predicting optimal radiation schedules, and subsequent experimental validation yielded novel radiation fractionation protocols that significantly improve survival in mice [74]. Due to the nature of the differential equation model, glioma stem cell division mechanisms were identified as contributing to improved survival, which will warrant further evaluation. Challenges for translating preclinical models into the clinic include the scalability from a total of 10 Gy radiation dose in one week for a mouse to the patient who receives routinely 50–60 Gy over many weeks, as well as the logistics of scheduling irregular hyperfractionated protocols vis-à-vis the increasing trend for stereotactic radiation.

**Figure 9.**
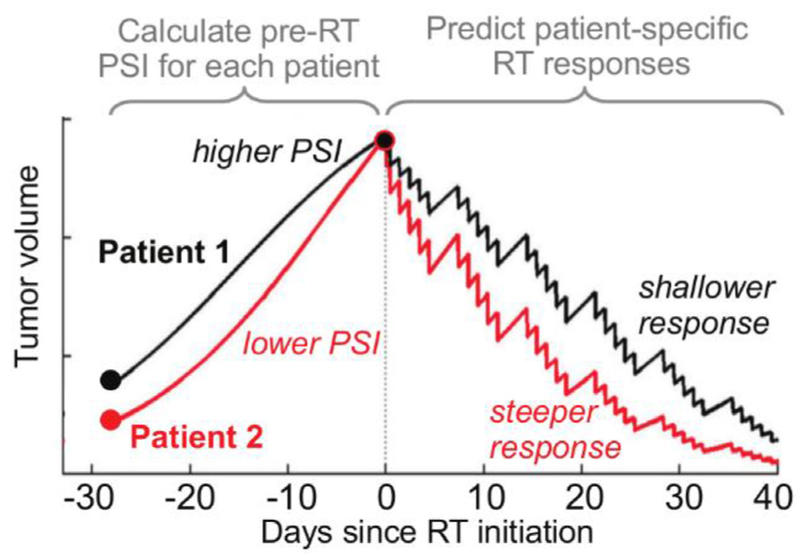
Pre-treatment tumor growth dynamics can be derived from volume measurements at diagnosis and treatment planning and used to calculate patient-specific PSI to predict RT responses.

Deriving an optimal total dose for individual patients was recently achieved by combining a molecular index of radiosensitivity (RSI), derived from gene expression analysis from pre-treatment biopsy tissue, with a mathematical model to derive a genomically adjusted radiation dose (GARD) [75]. To personalize dose fractionation, mathematical modeling of pre-treatment tumor growth between the diagnostic scan and radiation CT simulation has identified a proliferation saturation index (PSI, figure 9) [76, 77]. Based on PSI, patients can be non-randomly stratified into standard daily fractionation, hypofractionation, or twice-daily hyperfractionation protocols to achieve optimal tumor volume reduction. The estimation of GARD and PSI from, respectively, one or two patient-specific data points neglects the opportunity for multiple mathematical models to comparably simulate the data but potentially predict different dose and dose fractionations. Prospective clinical trials are necessary to fully evaluate the predictive power of mathematical model biomarkers.

The timeliest and arguably most challenging research question for radiation oncology is the optimal dose and dose fractionation to induce robust antitumor immunity, and how to optimally sequence immunotherapeutics to harness RT-induced immune responses. Few radiation protocols have been evaluated specifically for immune activation, and even fewer protocols have been studied with limited immunotherapy agents *in vivo*. Evaluating all possible treatment combinations at different timing and at different radiation and immunotherapy doses is experimentally and clinically impossible. Mathematical modeling of tumor-immune interactions trained to simulate available experimental and clinical studies and validated on independent data sets may provide a powerful tool for in silico trials of untested treatment combinations [78]. Numerical simulations, optimization theory, and high throughput machine learning approaches are poised to help identify promising synergistic protocols to maximize RT-induced anti-tumor immunity for local and systemic tumor control. Model-predicted therapies would still need to be prospectively validated and, even if unsuccessful, the newly derived data will help to iteratively improve the model to inform the next generation of clinical trials.

### Concluding remarks

RT is part of the therapy of more than half of all cancer patients, yet little research is directed to personalize radiation in the precision medicine era. Improving radiation treatment outcomes by a small margin will help more patients than the small target groups for novel clinical agents. We foresee a strong opportunity to integrate mathematical modeling with radiobiology and radiation oncology to address the immediate challenges for personalized RT. With a long history of successful physical models in radiation oncology, integration of mathematical modeling may be straight forward with a potentially large payoff.

### Acknowledgments

This work was supported in part by a pilot award from the NIH/NCI U54CA143970–05 (Physical Science Oncology Network (PSON)) ‘Cancer as a complex adaptive system’ and NIH grant U01 CA232137.

## 10. Evolutionary therapy

Alexander R A Anderson* and Robert A Gatenby*

Center of Excellence for Evolutionary Therapy, Integrated Mathematical Oncology Department, Moffitt Cancer Center, Tampa, FL 33612, United States of America

* These authors contributed equally, correspondence Alexander.

Anderson@Moffitt.org and Robert.Gatenby@Moffitt.org.

### Status

**Figure 10.**
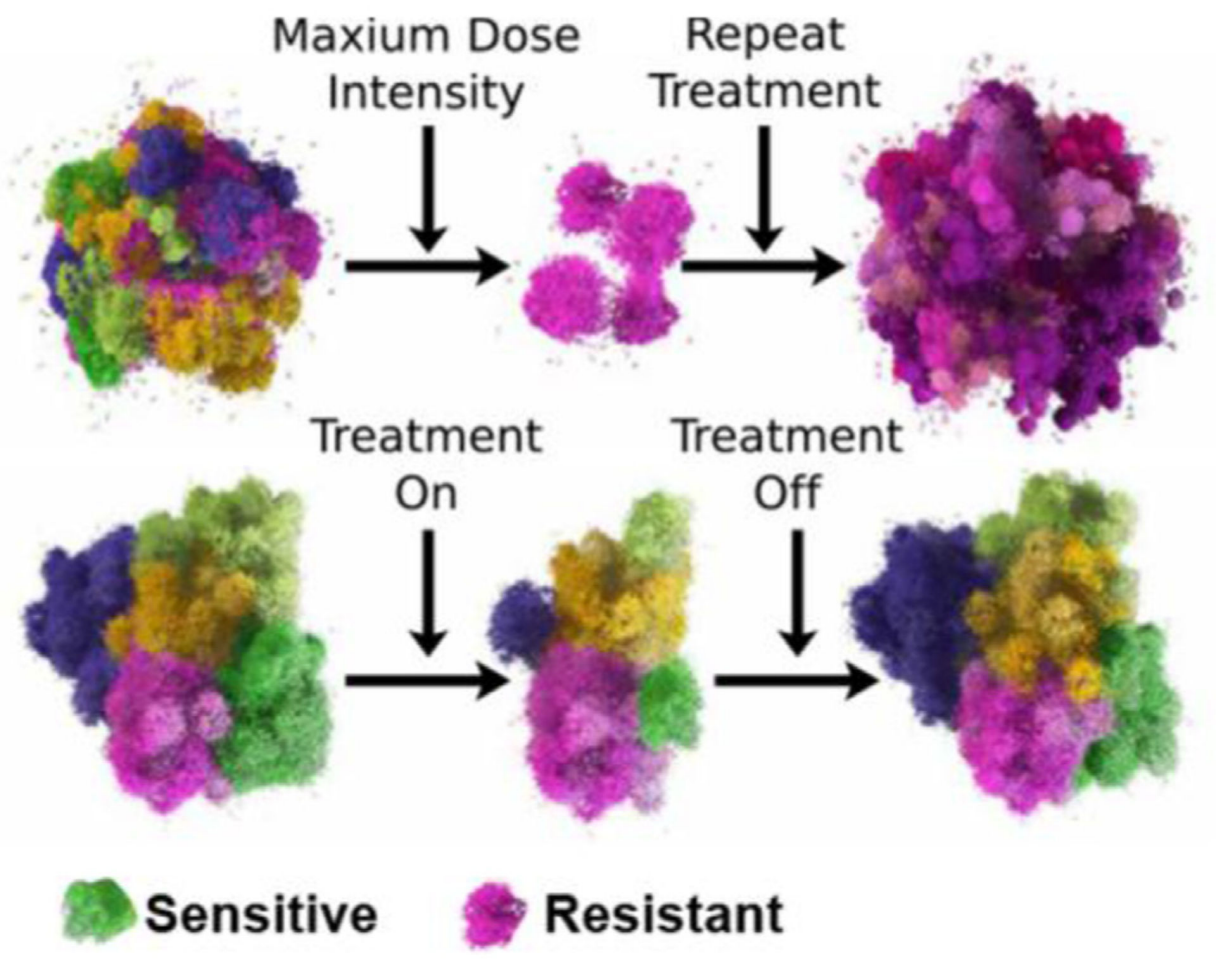
Conventional high dose therapy (top) maximally selects for resistant phenotypes (pink). Adaptive therapy (bottom) maintains a small population of cells that are sensitive to treatment. While the resistant cells survive, the cost of resistance renders them less fit in the absence of therapy. Thus, sensitive cells return when therapy is removed, suppressing growth of the resistant population.

Despite major advances in cancer therapies, most metastatic cancers remain fatal because tumor cells have a remarkable capacity to evolve drug resistance, both through genetic and non-genetic mechanisms. A common maxim in cancer treatment is to ‘hit hard and fast’ through dose-dense strategies that administer the highest possible drug dose in the shortest possible time period. The maximum tolerated dose (MTD) principle has been the standard-of-care for cancer treatment for several decades. In fact, all cancer drugs must go through a Phase I trial in which the MTD is established. The MTD strategy, however, only rarely cures patients with common disseminated cancers. An evolutionary flaw in this MTD strategy is the assumption that resistant populations are not present prior to therapy. It is now clear that resistant cancer cells are almost invariably present in the diverse cancer cell populations prior to treatment. This accounts for the consistent failure of MTD treatments to cure metastatic cancers, but the consequences are actually worse. MTD therapy, is designed to kill as many cancer cells as possible, although intuitively appealing, actually accelerates the emergence of resistant populations due to a well-recognized Darwinian phenomenon termed ‘competitive release’. As illustrated in figure 10, when high doses of drug are applied continuously, competitive release allows rapid emergence of resistant populations because of the combination of intense selection pressure and elimination of all potential ‘sensitive’ competitors. An alternative evolution-based strategy delivers the minimum effective dose (MED) that deliberately maintains a persistent drug-sensitive population. Treatment is then discontinued. Although the cancer population regrows, there is no selection for resistance so that it remains equally sensitive (figure 10). Through repeated treatment cycles, the tumor remains under control for extended periods of time. In mathematical models and early clinical trials, we have found the length of each cycle is highly patient-specific and can range from 4 months to 1.5 years, but very much depends on the specific cancer under consideration.

We have termed this approach ‘adaptive therapy’ and the eventual goal is to continuously adjust drugs, doses, and treatment schedules to prolong tumor control [79, 80].

Furthermore, adaptive therapy is only one example of a larger class of evolutionary-enlightened therapeutic approaches. Critically, as complex systems that span multiple spatial and temporal scales, these therapeutic perturbations of cancers often elicit non-linear dynamics so that outcomes can be predicted only using rigorously-defined, biologically-parameterized, and clinically-driven mathematical models. The Mathematical Oncology field must develop models that predict treatment responses in specific patients to enable the next generation of precision cancer medicine [81].

### Current and future challenges

Optimal treatment strategies, based on observed molecular targets, have been a focus of ‘precision oncology’ for some time. However, this approach has ignored a key piece of clinical reality—tumours are heterogeneous and evolve under the selection pressure of treatment. Therefore, even highly targeted and initially successful treatments almost inevitably fail as the cancer cells evolve adaptive strategies. Several mathematical techniques have been developed to model treatment outcomes. Evolutionary game theoretic approaches focus on the interactions between distinct subpopulations under different selection pressures in a frequency dependent manner [81, 84–86]. Ordinary Differential Equation models can capture population dynamics but often at the cost of over simplifying the interactions. More complex approaches, that explicitly include space [82, 83] or bridge multiple scales, have also been developed. However, a significant challenge with these approaches is how to calibrate them for a specific patient, since some of the parameters are abstract, e.g. fitness benefit or cost. Further, even for minimal models some parameters may be impossible to measure in a patient.

Assuming a model can be calibrated to a given patient, there is still a great deal of uncertainty regarding a specific fit since the model is gross simplification of reality. One approach to tackle this uncertainty is to develop multiple distinct models of the same tumour and generate an ensemble or consensus treatment plan, similar to hurricane prediction. Another is to deliberately consider all possible model parameter fits for a given patient as a cohort of patients that closely mimic the dynamics of the real patient. The successful *in silico* treatment strategies for the cohort then predict optimal therapy in the real patient. This ‘*Phase i* trial’ approach [87] allows us to both run an exhaustive array of all possible treatment options on the cohort but also allows for the cohort to be further refined as additional response data is obtained throughout the course of treatment. *Phase i* trials can also serve to bridge the divide between homogenous pre-clinical models and heterogeneous clinical reality as well as allowing for optimal strategies to be developed and tested before a drug ever reaches a patient.

This temporally changing treatment paradigm is a fundamental departure from the traditional fixed, one-size-fits-all strategy, and needs to be driven by a constant dialogue between model prediction and patient response. However, measuring and quantify patient tumour burden as well as intratumoural evolution during therapy using clinically-available patient data remains a major challenge.

### Advances in mathematics, technology, or data to meet challenges

There is a problem with big data in cancer. Its spatial scale is entirely molecular, it averages the properties of a large and heterogeneous population of cancer cells, and it is both an invasive and destructive procedure such that it is often only obtained at a single time point. While genome scale data can direct the choice of specific cancer drugs and potentially classify patients into different categories, it is mostly utilized in a correlative manner. If we hope to build mechanistic predictive mathematical models, most of ‘big data’ in cancer is the wrong data. It is not longitudinal, not spatial, only from one scale, averaged and homogeneous, not correlated or co-registered, and not analyzed within an appropriate micro-environmental context. There is an urgent need to gather the right data that will allow us to better define the cancer system and connect the scales of cancer, bridging genotype to phenotype, cell to tissue, organ to organism and individual to population. Because of the complex and dynamic nature of cancer it will not be sufficient to simply interpolate between data over these diverse spatial and temporal scales, rather we need to functionally integrate them through mathematical and computational models.

For a cancer patient, the reality of monitoring disease burden over time with sufficient frequency and resolution is currently infeasible. New technologies need to be developed that can readily monitor tumor burden in non-invasive and cost-effective ways that will directly facilitate the model prediction—treatment response loop of evolutionary therapy. The right data will depend on the potential treatments available, the specific cancer under consideration, and the constrained reality of clinical practice. Serum markers are currently our best candidates. However, not all cancers have a good surrogate for burden (e.g. Prostate Specific Antigen). Circulating DNA and circulating tumor cells are emerging areas of intense investigation and hold significant promise but these markers focus on molecular scale changes.

Thus, an additional challenge is linking mechanistic mathematical models to the genomic as well as the phenotypic scale. However, connecting the wealth of quantitative genomic information from a patient with functional cellular phenotypes remains an open question. Some progress is being made using machine learning approaches and mathematical models are emerging of cancer evolution at the genomic scale [88].

### Concluding remarks

There are currently 52 drugs approved for treatment of metastatic prostate cancer. Yet, every man who develops metastatic prostate cancer this year will not survive his disease. Throughout the past century, cancer therapy has focused entirely on the continuous development of new and more effective drugs. However, this enormous investment in time and resources has yet to significantly reduce the mortality rate of most common, adult metastatic cancers. Clearly, the drive to develop new and more effective cancer treatments is necessary. However, it is possible that the major impediment to improved outcomes in many cancers is not the absence of effective drugs but the absence of effective *strategies*. Thus, we view a key role for Mathematical Oncology is the development of patient calibrated mathematical models that integrate evolutionary first principles into cancer therapies to improve outcomes.

### Acknowledgments

The authors gratefully acknowledge ongoing support from the National Cancer Institute through both U54CA193489 and U01CA232382 and additional support from Moffitt’s Center of Excellence for Evolutionary Therapy.

## 11. Theoretical and experimental abstraction for understanding the fitness landscapes and evolutionary games of cancer

Artem Kaznatcheev^1,2^ and Peter Jeavons^1^

^1^ Department of Computer Science, University of Oxford, United Kingdom

^2^ Department of Translational Hematology & Oncology Research, Cleveland Clinic, OH, United States of America

### Status

Somatic *evolution* is now recognized as a central force in the initiation, progression, treatment, and management of cancer. This has opened a new front in the proverbial war on cancer: focusing on the *ecology* and *evolutionary biology* of cancer. On this new front, we are starting to deploy new kinds of mathematical machinery: fitness landscapes and evolutionary games.

A fitness landscape is a mathematical space where each point is a possible genotype or phenotype; two points are adjacent if they differ in a mutation or epimutation at a single locus; and each point has an associated fitness value. We often visualise evolutionary dynamics as ‘climbing up the hill’ of fitness values—although in high dimensional spaces it might be better to replace the mountain metaphor by a maze metaphor [89].

A central feature of fitness landscapes is the amount and kind of *interactions* between loci—such interaction is called epistasis. Synthetic lethality is a particularly important kind of epistasis for cancer cells. If there are mutations at two loci which each change the fitness in one direction when they occur on their own, but in the opposite direction when they both occur together—either bad + bad = good, or good + good = bad—then the landscape is said to have *reciprocal sign epistasis*. It has been shown that any fitness landscape with more than one local peak must have reciprocal sign epistasis.

Fitness landscapes conceptualize *fitness* as a single scalar value—a number. But a scalar can only express cell-autonomous effects, where fitness is inherent to the properties of a single cell. But cancer displays important non-cell-autonomous effects that allow fitness to depend on a cell’s micro-environmental context, including frequency of other cell types [90, 91]. To accommodate this, evolutionary game theory (EGT) views these cell types as strategies, and models fitness as a *function*, which depends on the abundance of strategies in the population. On the surface, the games perspective is more expressive, since scalars can be represented as constant functions.

But as always we pay for greater expressiveness by a loss of analysis techniques. For example, when dealing with fitness landscapes, we can often consider the strong-selection weak-mutation limit, which allows us to replace a population by a single point in the landscape. In the case of evolutionary games, such an approximation is unreasonable since it would eliminate the very ecological interactions that EGT aims to study. This means that the strategy space that can be analysed in an evolutionary game is usually much smaller than the genotype/phenotype space considered in a fitness landscape. Typical EGT studies consider just a handful of strategies (most often just two [90, 92, 93]), while fitness landscapes start at dozens of genotypes and go up to tens of thousands (or even hyper-astronomical numbers of genotypes in theoretical work [89]). However, there is ongoing work on *adaptive landscapes* that aims to combine the strengths of fitness landscapes and game theory.

Game theory models have so far had more direct impact in oncology than fitness landscape models. The standard approach has been to develop a game theory model from the bottom up, starting from a reasonable reductive grounding and adding micro-dynamic details. This is in keeping with the reduction-ist tactics used on the old cell and molecular biology front. For example, Basanta *et al* [92] studied motility in cancer by defining two intuitive strategies: Go versus Grow. The first model included no spatial aspects; later work built on this by adding minimal spatial effects and considering the heterogeneity of spatial structure in a tumour [93]. This progression to more complicated and detailed models is a common pattern among EGT models in oncology. The other common aspect is that the games rely on biological or clinical intuition; the exact game parameters are seldom measured. This EGT perspective has helped oncologists to express a number of interesting theoretical consequences of non-cell autonomous processes, but has only recently started to be translated into direct experimental work.

### Current and future challenges

Compared to EGT, fitness landscapes have not been as extensively used beyond mental models in oncology. But they have been central to work in understanding evolution of *Escherichia coli* and yeast. The most notable example might be Lenski’s long-term evolution experiment with *E. coli* that has been propagating 12 initially identical populations for over 70 000 generations since 24 February 1988. Another example is the study of the evolution of drug resistance in microbes [94], which has direct parallels to evolution of resistance in cancer.

The key difficulty in developing fitness landscape models for oncology is that cancer cells are more complex, and oncological experimental systems are less well-controlled, than their microbial counterparts. In particular, micro-dynamical foundations of somatic evolution, reprogramming of human cells, and *in vitro* mutation operators are less well understood. The tactic from the old front of the molecular and cell biology of cancer would be to study and classify these micro-dynamics in more and more detail. On the new front of somatic evolution, we have a more promising tactic—*abstraction*.

For a computer scientist, abstraction is a way to hide the complexity of a computer system. It is a way to make programs that can be used and re-used without having to re-write all the code for each new computer. In this sense an algorithm is an abstraction of the actual sequence of bit flips that carry out the physical process that is computation. To turn it around: the physical process carried out by your computer is then an implementation of some abstract algorithm. Abstraction and implementation are in some sense dual to each other.

Using this kind of abstraction, the tools of theoretical computer science can be introduced into oncology to reason rigorously about our models without knowing all the details of the implementation. For example, using computational complexity Kaznatcheev [89] conclude that there are ‘hard’ fitness landscapes where no evolutionary dynamic can find local fitness optima in polynomial time. This is an abstraction over the micro-dynamical basis of evolution. If such fitness landscapes occur in tumours, then—no matter how complicated the (re)programming of the cell or how strange and biased the mutation operator—the cancer cells will not reach a local fitness peak and thus will always provide a moving target for therapy. It becomes an empirical problem to find out if such landscapes occur in cancer. And it becomes a theoretical problem to discover other abstract properties of fitness landscapes that are robust under any implementation of the evolutionary micro-dynamic.

Abstract objects or processes are multiply-realizable by several concrete objects or processes. The concrete objects might differ from each other in various ways, but if the implementations are ‘correct’ then the ways in which they differ are irrelevant to the abstraction. The abstraction is less detailed than the implementation but captures essential features precisely.

It might seem like connecting more closely to experiment must always make a model less abstract. But this is not always the case: the act of measurement itself can be a way to abstract. This is achieved with phenomenological or effective (instead of reductive) theories, and is easiest to illustrate in a game theory model. For example, Kaznatcheev *et al* [91] developed a game assay based on the frequency and growth rate of types (as opposed to the more standard view of fitness of tokens or specific individuals). The focus on abstract types lets us absorb all the details of spatial structure, interaction length-scales, reproductive strategies, etc into the measurement of the type fitness [91, 95]. It is nature that figures out the particular computation that transforms token fitness into type fitness (see figure 11 for 3 examples) and we do not need to know it once we are working at the level of the abstract effective game: the abstract measurement is enough to derive the predictions of the model. A downside, of course, is that we cannot describe the specific way token fitness is translated into type fitness in our system. But future work can push the abstraction down, so that more details of the implementation—such as the effects of spatial structure—can be extracted [95]. This approach has already led to both new theoretical frameworks and new experimental techniques for analysing evolutionary games in microscopic cancer systems [91, 95], but more focus on effective theories is needed—especially for fitness landscapes.

### Advances in mathematics, technology, or data to meet challenges

Although games can be viewed as a generalization of fitness landscapes, the game assay can only be used for a small strategy space. The size of a fitness landscape, however, is exponential in the number of loci, so it quickly becomes impossible to explicitly measure and record the fitness of every single possible genotype (or strategy). This barrier cannot be overcome by better technology or experiments. Instead, we need to focus on fitness landscape models with compact representations that are learnable from a polynomial number of samples. These compact representations are akin to rules for genotype-phenotype maps (where the relevant phenotype is fitness): they specify a rule for computing the fitness value from the genotype (or at least the relative order of fitness), instead of explicitly storing a fitness value for each genotype.

To find compact representations it is tempting to turn to existing representations like oncogenetic trees or cancer progression models (CBN and CARPI, in particular), but these models cannot express the reciprocal sign epistasis that makes for interesting fitness landscapes [96]. Instead, we need models that can represent gene-interaction networks with low-order epistasis (a classic example would be spin glasses) [89, 97]. These gene-interaction networks can express reciprocal sign epistasis. Most importantly, such networks can be inferred from polynomially-sized local fitness landscapes: from fitness values just a couple of mutations away from a wildtype, instead of measuring every possible combination of mutations. Such local landscapes have been measured in yeast [98], similar measurement techniques need to be developed for cancer systems.

### Concluding remarks

On this new front in the war on cancer that was opened by somatic evolution, we not only need new mathematical machinery, like fitness landscapes and evolutionary games, but also new tactics, like abstraction. To handle complex systems like cancer where we do not know the detailed evolutionary micro-dynamics we need abstract models that extend the tools and techniques developed in microbiology and ecology to handle multiple-realizability. The lesson from computer science is that rigorous abstraction provides great theoretical power. And experimentally, we need to recognize that abstract is not the opposite of empirical. Abstract models can serve as phenomenological (or ‘effective’) theories that use carefully defined measurements to account for multiple-realizability from unknown micro-dynamic details. Developing this approach will allow us to better use the mathematical machinery of fitness landscapes and evolutionary games for understanding and treating cancer.

## 12. Evolutionary game theory and fitness landscapes as frameworks for predicting and preventing drug resistance in cancer

Nikhil Krishnan^1^, Julia Pelesko^1^, Raoul R Wadhwa^1^, Nara Yoon^2^, Artem Kaznatcheev^2,3^, Daniel Nichol^4^, Andriy Marusyk^5^, Michael Hinczewski^6^ and Jacob G Scott^2^

^1^ School of Medicine, Case Western Reserve University, Cleveland, OH 44106, United States of America

^2^ Department of Translational Hematology & Oncology Research, Cleveland Clinic, Cleveland, OH 44195, United States of America

^3^ Department of Computer Science, University of Oxford, Oxford OX1 3Q, United Kingdom

^4^ The Institute of Cancer Research, London SM2 5NG, United Kingdom

^5^ The Department of Cancer Physiology, H. Lee Moffitt Cancer Center & Research Institute, Tampa, FL 33647, United States of America

^6^ Department of Physics, Case Western Reserve University, Cleveland, OH 44106, United States of America

### Status

Predicting, detecting, and preventing drug resistance is an enduring challenge in clinical oncology. The initial development of targeted therapies provided hope that targeting the unique molecular drivers of a tumour would allow for more precise treatment with fewer clinical side effects. However, in many cases, after a transient killing of tumour cells, drug resistance leads to therapeutic failure. Indeed, cancer is an intrinsically evolutionary process in which cell populations comprising the tumour adapt and evolve in response to the selective pressures placed upon them, particularly anti-cancer therapies [99]. Cancer treatment is subject to the same dilemma of drug resistance that has complicated the treatment of infections with antibiotics [100]. In both cancer and infectious diseases, drugs apply a selective pressure that yields higher frequencies of phenotypes better-suited to survive in their environment. Even the most advanced therapies are ultimately at the mercy of the Darwinian principles of evolution.

**Figure 12.**
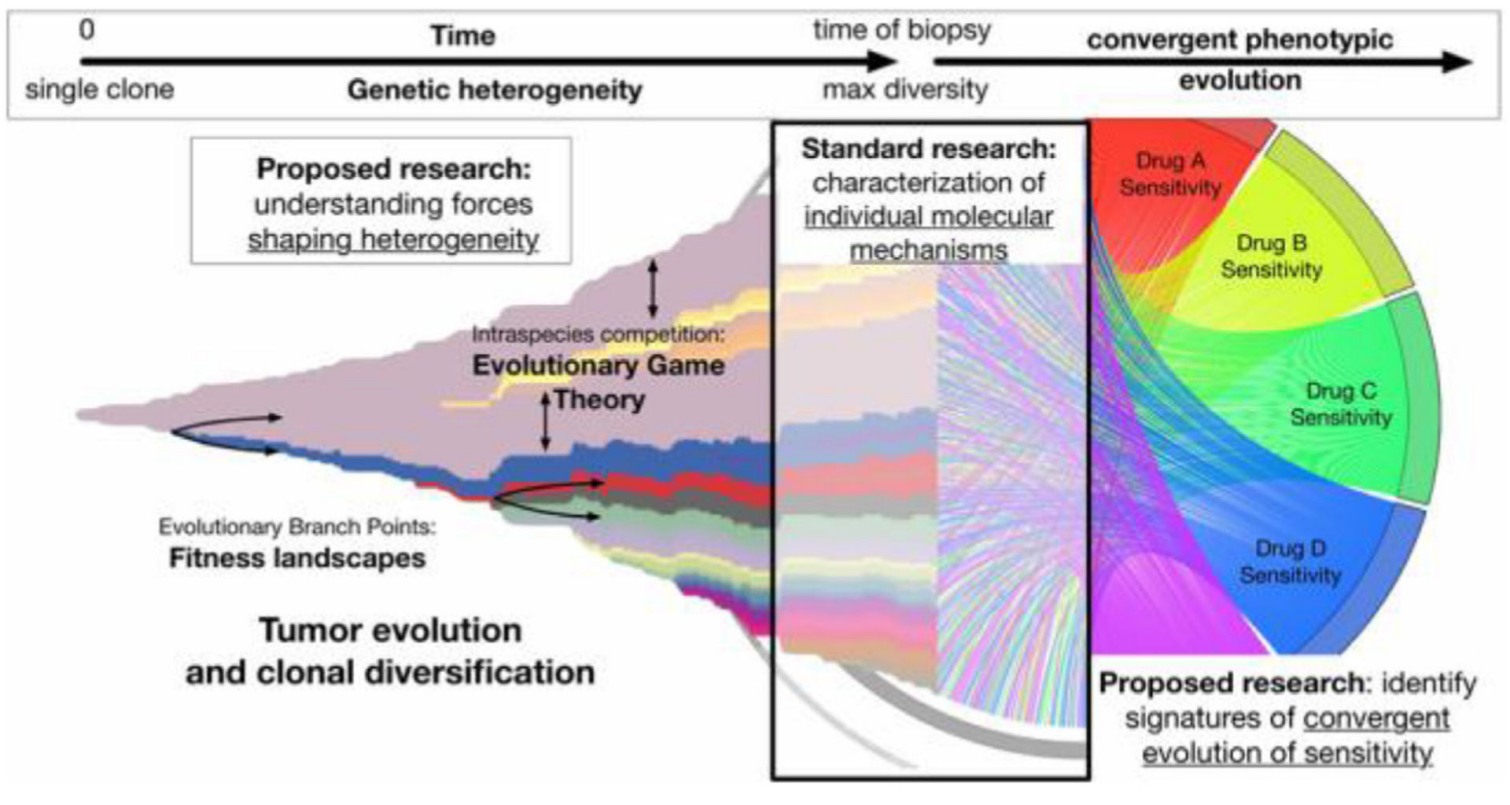
Correspondence of research strategies to the process of tumour evolution: Tumour heterogeneity is driven by clonal populations traversing evolutionary trajectories, the interactions between them, and the diversification that results. The milieu of molecular mechanisms that can be observed at the time of biopsy potentially confers finite drug sensitivity phenotypes.

When it comes to discerning the separated time scales of this evolution of drug resistance, we have found a singularly focused strategy to be insufficient. As we strive for our work to tangibly affect clinical care, the fields of population genetics, evolutionary and adaptive dynamics, and statistical physics may offer models and tools that provide an improved understanding of the temporal dynamics of resistance evolution and the necessary timescales of potential interventions (figure 12). Two specific examples from the emerging field of evolutionary therapy have demonstrated how the marriage of disparate theoretical and experimental tools have brought us closer toward the goal of evolutionarily informed therapy.

One body of work utilizes evolutionary game theory (EGT) to deliver insights into the qualitative relationships between the ‘players’ in the tumour micro-environment. Another complimentary strategy models cancer evolutionary dynamics on fitness landscapes and is more amenable to genomic sequencing data and addressing tumour heterogeneity.

While we only discuss two models here with several unanswered questions standing in the way of their full potential, we believe they represent research strategies that are ripe for the interdisciplinary scientific groups and institutes of today and beyond.

### Current and future challenges

Evolutionary game dynamics have been found to describe diverse phenomena in cancer ranging from IGF-II production in pancreatic tumours to tumour-stroma interactions in prostate cancer [101, 102]. While these models may yield insights into the qualitative relationships between the players in the tumour micro-environment, it is unclear if the evolutionary game modelled in different cancers is applicable to other cancers, or even every patient with a given cancer. Furthermore, the payoff matrix used to describe the games played in most models of evolutionary games is not empirically derived, but rather inferred from clinical intuition. It seems that a means of measuring these micro-dynamic interactions among tumour cells in any given tumour is necessary.

An additional drawback of the EGT formalism is the difficulty in using it to characterize the vast heterogeneity and mutational activity that are critical components of the evolution of drug resistance. Current biological methodologies may only allow for the study of interactions between a few cell types. Studying fitness landscapes may address this issue of accounting for tumour heterogeneity. Fitness landscapes are a genotype-phenotype map, in which each allele is assigned a corresponding fitness and set of neigh-bouring alleles. Suggested first by Sewall Wright, in the canonical model of fitness landscapes, each allele can be represented as a string of ones and zeros corresponding to mutated and wild-type alleles, respectively. The entire landscape can be represented as a network of these bit strings with a hypercubic topology. Each node has its own fitness, and evolution proceeds as a biased random walk through the winding maze of genotype space, as it tends toward the most fit geno-types available to it [103].

**Figure 13.**
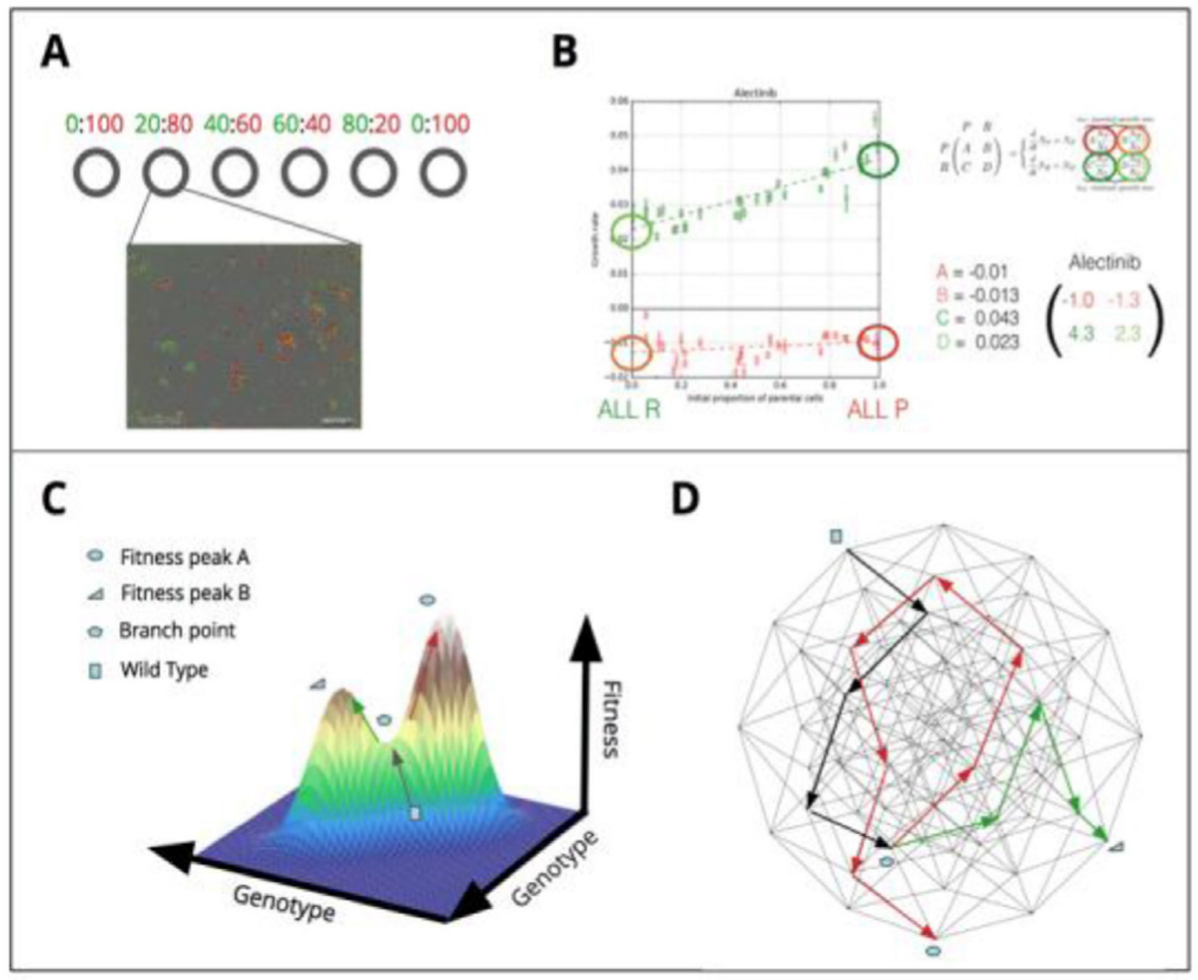
(A) Diagram of experimental setup, with varying proportions up drug sensitive (red) and resistant (green) cells. (B) Parameterization of the two-player game matrix, given the linear relationship between growth rate of drug resistant cells and proportion of seeded sensitive cells. (C) 2D representation of a fitness landscape in which the *x*-*y* plane represents genotype, and the landscape height represents fitness. From a single starting point of the wild-type genotype, diverging evolutionary trajectories emerge from saddle points, ending at multiple possible local fitness optima. (D) A six locus landscape drawn as a directed graph, in which each node is a genotype and each edge represents an evolutionary path between them. Arrows illustrate potential paths through multi-dimensional genotype space, toward local fitness optima as shown in panel C.

Any environmental condition (i.e. cancer therapies) can apply a selective pressure to a population (i.e. a tumour) that specifies a corresponding fitness function over the domain of genotype space (figures 13(C) and (D)).

For fitness landscapes to be clinically useful there are challenges to be overcome. These include, but are not limited to: measurement, definition, and inference of fitness landscapes in clinically-relevant contexts and determination of appropriate time-scales of possible interventions.

### Advances in mathematics, technology, or data to meet challenges

We have recently suggested one method to experimentally parametrize EGT matrices: the evolutionary game assay. We observed in our cultures of alectinib resistant and sensitive lung cancer cells a linear frequency dependence of resistant cell growth rate on the proportion of sensitive cells, allowing us to represent the interactions in the four conditions studied as a two-strategy matrix game and infer the games played [91]. We showed that in our experimental system of drug resistant and sensitive cells, in the presence of alectinib and stromal cells, a qualitatively different game is being played (figures 13(A) and (B)).

Our evolutionary game assay is designed specifically to capture the ‘effective’ game being played. Along with work aimed at characterizing how spatial information implies information about tumour-environment interactions, our assay to empirically measure games may be used to design clinical trials in any cancer type [82, 104].

To address our game theory assay’s inadequacy at accounting for tumour genetic heterogeneity, we turned to fitness landscapes. Our theoretical work has revealed an interesting feature of an evolving population subjected to sequential drugs: evolution through these sequential drug landscapes is irreversible. Given complete information of each fitness landscape, this feature allows for what we call ‘steering’ using drug sequences, that is, using drugs to purposely *push* the cancer cell population to states of sensitivity from which resistance is less likely to be reached [94]. This steering of evolutionary dynamics reveals yet another strategy for evolutionary therapy. We propose that with the advent of computational methods to infer fitness landscapes on temporal and genomic scales, one could design patient-specific steering drug regimens to minimize drug resistance. We have performed further high throughput evolution experiments that suggest its feasibility [105].

Addressing the previously described challenges of utilizing fitness landscapes to optimize treatment sequence regimens may require exploiting the close analogy between evolutionary fitness landscapes and energy landscapes in other contexts. Techniques from statistical physics that allow for the inference of properties of energy landscapes through experimental probes or protocols that vary the stochastic dynamics of these landscapes over time, can potentially be adapted to the evolutionary context.

### Concluding remarks

Our EGT assay and evolutionary steering method provide examples of how understanding the evolutionary dynamics of cancer through both theoretical and molecular biological tools can lead to more effective treatments for patients. In the future, this can lead to treatment sequence regimens optimized to steer a tumor away from resistance and treatments that alter the tumor microenvironment to modulate the evolutionary game being ‘played’ and decrease the resulting proportion of resistant cells. As both frameworks highlighted here have their advantages and drawbacks, it is only by exploring them in concert and considering how they may complement each other that we can apply them most effectively. Fitness landscapes’ failure to address the tumour micro-environment, for instance, motivations exploration of EGT, which is well suited to examine such interactions. To look forward we must look backward, extending and blending these readily available tools. The application of these well-established theories, methodologies, and drugs, re-imagined and reconsidered in novel ways is a promising path forward to overcome therapeutic resistance in cancer.

## Figures and Tables

**Figure 8.**
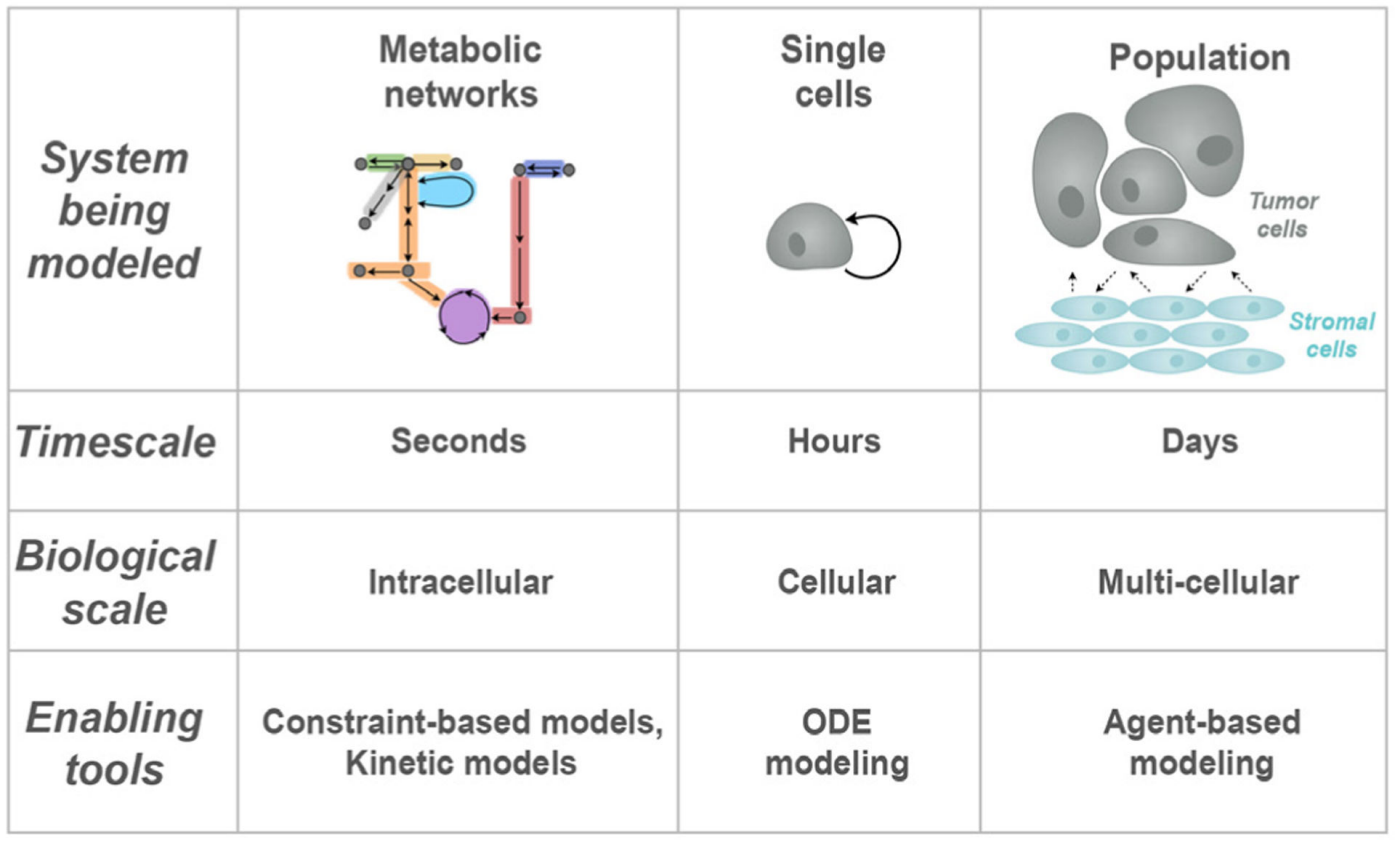
Schematic of relevant systems investigated when modelling cancer metabolism.

**Figure 11.**
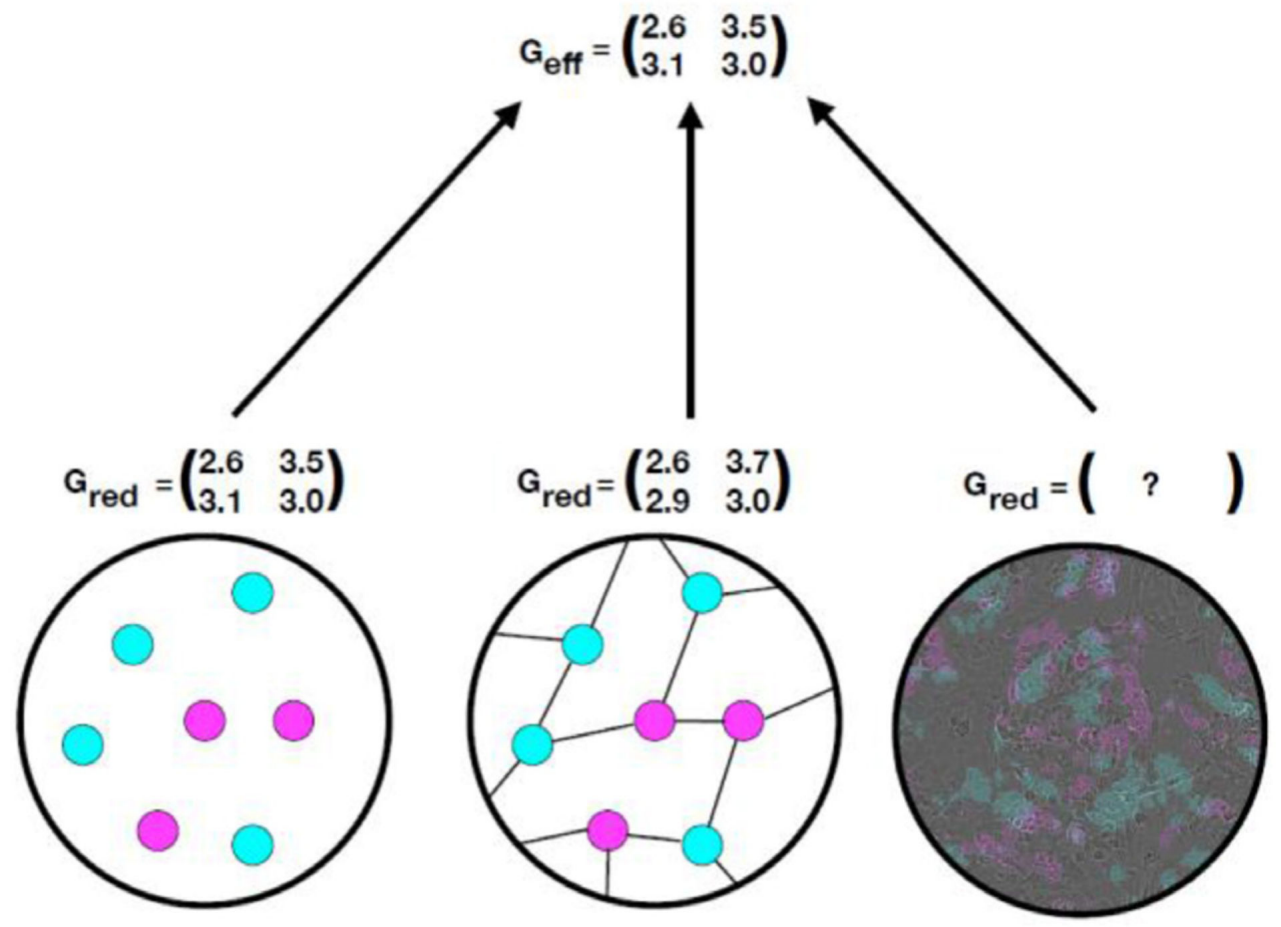
The same effective game [95] implemented by three different population structures and reductive games; from left to right: inviscid population, random 3-regular graph, experimental *in vitro* non-small-cell lung cancer [91].
